# Maize Anther Development Involves Translated Open Reading Frames From 3′ Untranslated Regions

**DOI:** 10.1002/advs.202523401

**Published:** 2026-02-20

**Authors:** Chunyu Wang, Yuechao Wu, Shunxi Wang, Han Liu, Siqi Jiang, Yingjia Han, Liuji Wu, Tao Zhang, Mei Zhang

**Affiliations:** ^1^ State Key Laboratory of Forage Breeding‐by‐Design and Utilization Institute of Botany Chinese Academy of Sciences Beijing China; ^2^ University of Chinese Academy of Sciences Beijing China; ^3^ Chengdu Institute of Biology Chinese Academy of Sciences Chengdu China; ^4^ State Key Laboratory of High‐Efficiency Production of Wheat‐Maize Double Cropping College of Agronomy Henan Agricultural University Zhengzhou China; ^5^ College of Plant Science and Technology Beijing University of Agriculture Beijing China; ^6^ China National Botanical Garden Beijing China

**Keywords:** 3′ untranslated region, anther development, anther, maize, photosynthesis, translational regulation

## Abstract

Few studies have explored the functions of peptides encoded by open reading frames (ORFs) in annotated noncoding regions, particularly the 3′ untranslated regions (3′ UTRs). Although translated ORFs in 5′ UTRs (5′ ORFs) have been shown to inhibit translation of their corresponding main ORFs (mORFs), the roles of 3′ ORFs remain poorly understood. This study analyzes translational regulation across ten developmental stages of maize (*Zea mays*) anthers and finds that peptides translated from 5′ or 3′ ORFs can represent misidentified isoforms, with 3′ ORFs potentially linked to anther sterility. Notably, the cloned *APV1* locus, whose mutation causes male sterility, exemplifies this relationship. Genome‐wide translation profiles further reveal enrichment of photosynthesis‐related genes during Phase III (the binucleate microspore stage). The presence of stomata and the observed low electron transport rate and net photosynthetic rate suggest that anthers assimilate CO_2_ with limited photosynthetic efficiency via a pathway distinct from typical C_4_ photosynthesis. Overall, this study identifies 3′ ORFs as potential targets for generating male‐sterile maize lines and provides new insights into anther photosynthetic activity.

## Graphical abstract

This study examined the translation activity across ten stages of maize anther development, integrating transcriptome and small‐peptide data. It linked mutations in translated 3' ORFs to anther sterility, exemplified by *Zm00001eb417050*_3' ORF (known as *APV1*). In addition, the data indicated that maize anthers could assimilate CO_2_ through the stomata in the connective tissue and the surrounding area.

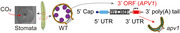

## 1 Introduction

Male‐sterile lines are a crucial component of maize (*Zea mays* L.) breeding and seed production. Understanding the development of the male reproductive organ, the anther, is essential for creating new male‐sterile lines [1]. Maize anther development can be divided into 14 stages [2, 3, 4, 5]. Transcriptome profiling has grouped these stages into four major phases: cell fate and differentiation (Phase I, 0.2–1.3 mm), meiosis (Phase II, 1.5–3.0 mm), pollen maturation (Phase III, 4.0 mm), and mature pollen (Phase IV) [6].

Maize anthers have highly complex transcriptomes. Pre‐meiotic anthers (Phase I) express about 30 000 genes, which represent nearly three‐quarters of all annotated maize genes [7, 8, 9, 10, 11, 12]. A spatiotemporal transcriptional network constructed from ten developmental stages revealed dynamic changes in gene expression throughout anther development [6]. However, while transcriptional regulation has been extensively studied, translational regulation during maize anther development remains poorly understood. The peptide‐associated translatome has been characterized only in part, and only a few studies have used mass spectrometry (MS) to examine protein expression during early anther development [8, 13, 14, 15, 16, 17].

Ribosome profiling (Ribo‐seq) is a powerful method for studying active mRNA translation at single‐codon resolution [18]. In plants such as rice (*Oryza sativa*) [19], soybean (*Glycine max*) [20], wheat (*Triticum aestivum*) [21], and *Arabidopsis thaliana* [22], Ribo‐seq has highlighted the importance of translational regulation in developmental processes. Notably, Ribo‐seq revealed regulatory roles of open reading frames (ORFs) within regions of protein‐coding genes that are generally considered untranslated [23, 24, 25, 26]. A study in maize seedlings showed that translated upstream ORFs (uORFs) can inhibit translation of the main ORF (mORF) of the gene [27]. For example, a uORF in the 5′ untranslated region (UTR) of the maize *R* gene inhibits translation of its mORF [28]. In addition, *Enod40*, whose 5′ end (region I) contains ORFs encoding 12‐ and 24‐amino acid peptides (peptides A and B), regulates sucrose use in alfalfa (*Medicago sativa*) and influences root nodule organogenesis in soybean (*G. max* cv. Jutro) and *Medicago truncatula* (cultivar R108) [29, 30, 31]. A recent study in *Arabidopsis* suggested that Ribo‐seq can misidentify unannotated ORFs as uORFs within 5′ UTRs of genes that are not properly annotated [32]. Beyond the 5′ UTR, numerous translated ORFs have also been detected in 3′ UTRs [19, 25, 27, 33, 34, 35, 36]. However, the functions of ORFs in 3′ UTRs remain poorly understood. These studies indicate that additional, functionally uncharacterized ORFs may exist within both the 5′ and 3′ UTRs of known protein‐coding genes.

In this study, we combined RNA‐seq and Ribo‐seq data to characterize translational regulation at ten key stages of maize anther development. We found that ORFs overlapping UTRs can be transcribed from independent isoforms of protein‐coding genes around, and that translation of 5′ UTRs can negatively affect translation of downstream mORFs. In addition, independent isoforms translated by 3′ ORFs were correlated with maize sterility, exemplified by the *APV1* locus. Moreover, our data showed that maize anthers have fewer stomata and lower electron transport rate and net photosynthetic rate than mature leaves, suggesting reduced photosynthetic efficiency. Overall, this study provides new insights into the roles of 3′ UTR–translated peptides in plant development and offers potential strategies for producing male‐sterile lines for maize breeding.

## 2 Results

### 2.1 Phase Division of Maize Anther Development

**FIGURE 1:**
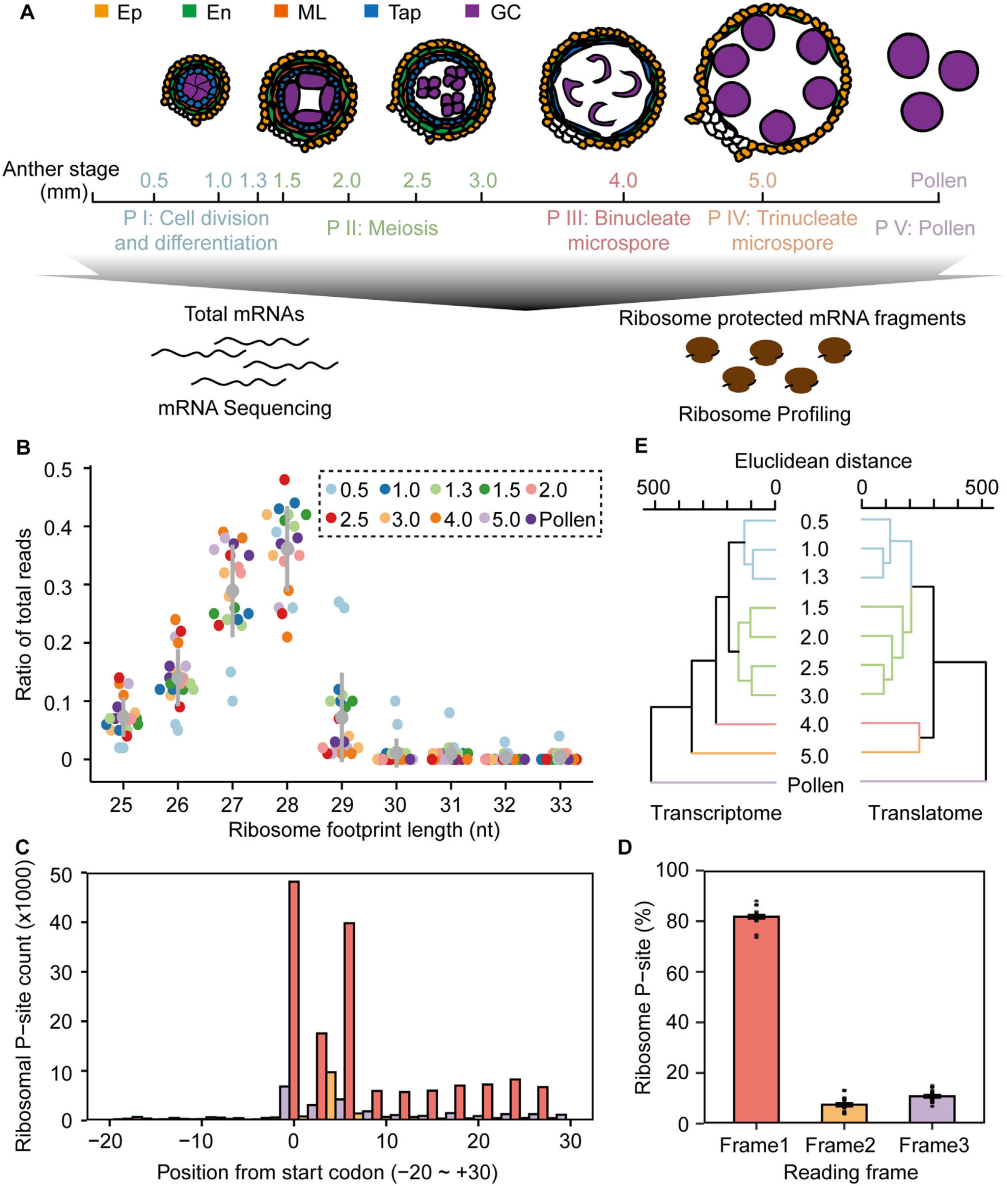
Ribosome profiling and RNA sequencing reveal translational dynamics during maize anther development. (A) Overview of the experimental design. Transcriptome and translatome analyses were performed on maize anthers from ten developmental stages, including nine stages with anther lengths from 0.5 to 5.0 mm and the final stage representing mature pollen. Ep, epidermis; En, endothecium; ML, middle layer; Tap, tapetum; GC, germinal cells; P, phase. (B) Size distribution of ribosome footprints. nt, nucleotide. Most reads were distributed between 25 and 30 bp. (C) Positions of P‐sites within the first 50 nt of annotated ORFs, based on offset‐corrected Ribo‐seq reads from 2.5‐mm anthers (replicate 1). (D) Percentage of ribosome footprints in each reading frame across the ten developmental stages of maize anthers. (E) Hierarchical clustering of Ribo‐seq and RNA‐seq data from the ten anther developmental stages. The dendrogram was constructed using Euclidean distance to evaluate dissimilarity between transcriptome and translatome gene expression profiles. Numbers indicate anther lengths (mm) at each stage. Colored lines correspond to the five developmental phases shown in panel (A).

To investigate the dynamics of gene translation during maize anther development, we systematically analyzed Ribo‐seq and RNA sequencing (RNA‐seq) data from tissues representing ten developmental stages of anthers (Figure 1A) [37]. Ribosomal footprints exhibited the expected size distribution of 25–33 bp (Figure 1B) and mapped primarily to coding sequences (CDSs) with strong three‐nucleotide periodicity, a hallmark of active ribosomal translation (Figure 1C,D; Figure S1). These patterns are consistent with those reported for maize seedlings [27], *Arabidopsis* [22], rice [19], and other plant species [21, 38, 39]. Principal component analysis (PCA) confirmed that biological replicates clustered closely, whereas samples from different developmental stages were clearly separated (Figure S2). Combined with the > 96% correlation among replicates (Figure S3), these findings indicate that both the transcriptome and translatome datasets are highly reproducible and suitable for further analysis.

We next calculated the numbers of transcribed and translated genes using a cutoff of Fragments Per Kilobase of exon model per Million mapped fragments based on RNA‐seq (FPKM) or Reads Per Kilobase per Million mapped reads based on Ribo‐seq (RPKM) ≥1 (Data S1). Across all ten developmental stages, the number of translated genes (approximately 18 000 per anther sample and ∼7000 in pollen) was lower than the number of transcribed genes (approximately 22 000 per anther sample and ∼8000 in pollen) (Figure S4A). Overall, about 80% of genes transcribed in anthers were detected as translated, and about 95% of translated genes were supported by transcriptomic data. These proportions were lower in pollen, with 67% of the transcriptome and 76% of the translatome represented (Figure S4A). The genes detected by translatome (Ribo‐seq) but not by transcriptome are likely to be expressed but have mRNA expression levels below the robust detection threshold in the profiled stage (Figure S4B,C). Moreover, approximately one‐third of genes detected at both the transcriptional and translational levels were also identified in proteomic data from 1.0‐ and 2.0‐mm anthers and pollen, as published by Walley et al. (2016) (Data S1) [17].

Based on these results, we divided the ten anther developmental stages into five phases (Figure 1E; Figure S2C,D). The divisions for Phases I–III follow Han et al. (2022), and mature pollen is considered the final phase [6]. We added an additional 5.0‐mm anther stage (designated Phase IV, the trinucleate microspore stage) to cover the entire process of anther development.

### 2.2 Comparison of the Transcriptome and Translatome During Anther Development

**FIGURE 2:**
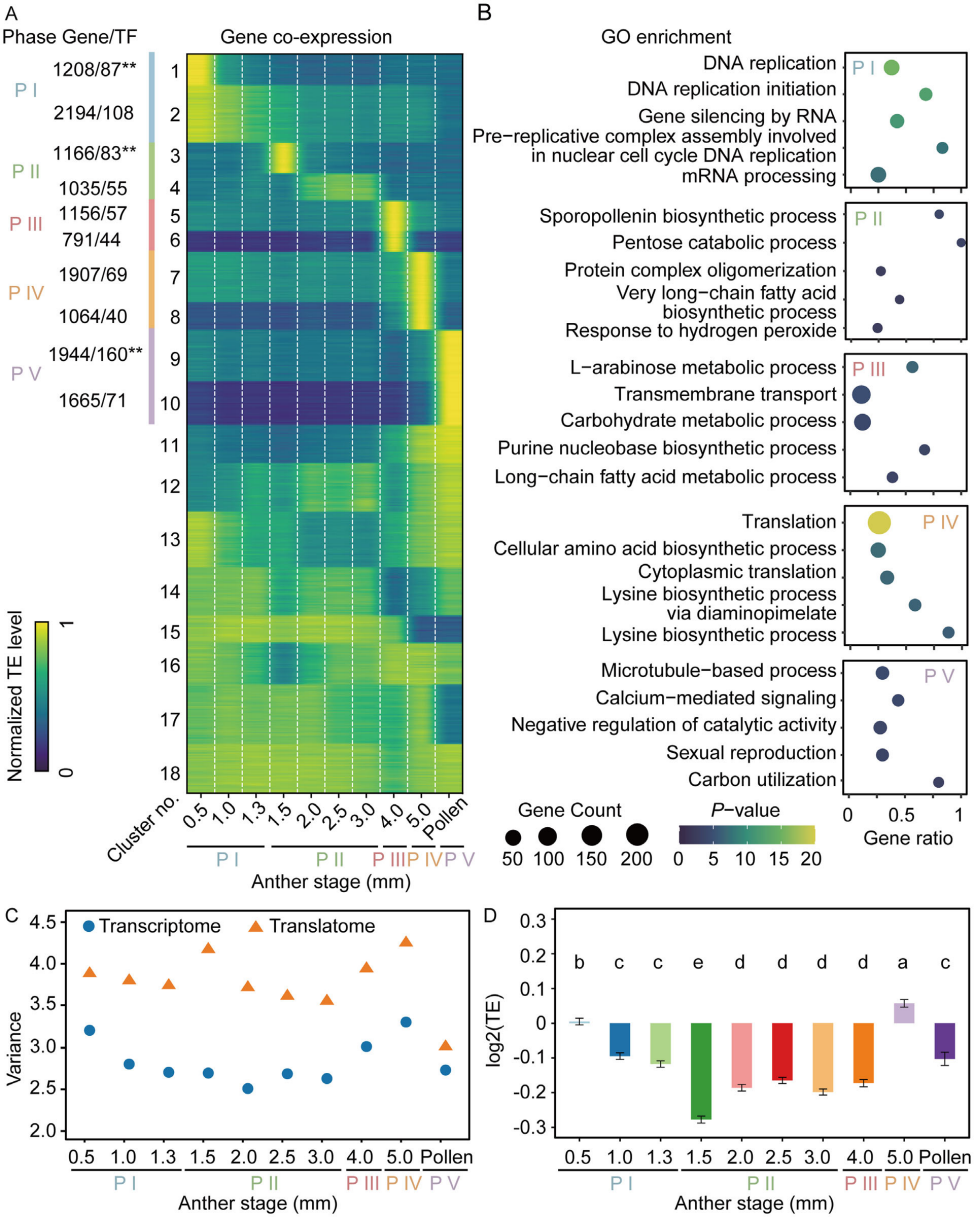
Heatmap and gene ontology analysis of gene coexpression clusters based on translation efficiency. (A) Gene coexpression analysis of translation efficiency (TE). TE values for all genes were normalized to the maximum TE value across the ten developmental stages. Numbers along the heatmap indicate the number of genes and transcription factors (TFs) within each cluster. The number to the left of the slash represents the number of genes, and the number to the right represents the number of TFs. Asterisks denote significant TF enrichment. Adjusted *p* values were calculated using the hypergeometric distribution test (^*^ *p* <0.05; ^**^ *p* <0.01). (B) Gene Ontology (GO) enrichment analysis of the first ten phase‐specific clusters. The x‐axis represents the gene ratio (the proportion of genes associated with each GO term). Dot size indicates the number of genes, and color represents the adjusted *p* value. Adjusted *p* values were calculated using the hypergeometric distribution test. (C) Distribution of expression levels in the translatome (orange, Ribo‐seq) and transcriptome (blue, RNA‐seq). Expression variation, quantified as variance across genes of log_2_(FPKM_RNA‐seq_ or RPKM_Ribo‐seq_ + 1)‐transformed values, is indicated. (D) Bar plot showing genome‐wide gene TE across all ten stages. The phase divisions of anther development are shown in Figure 1. P, phase.

To characterize the translational expression dynamics of genes during maize anther development, we performed gene coexpression analysis based on translation efficiency (TE), defined as the ratio of ribosome footprints to mRNA abundance. According to their TEs, genes were grouped into 18 coexpression clusters (Figure 2A). Gene ontology (GO) enrichment analysis of the first ten phase‐specific clusters revealed distinct biological functions associated with different developmental phases (Figure 2B). Notably, in Phase IV (5.0‐mm anther), genes related to “translation” were significantly enriched (*p* = 4.9 × 10^−42^; Figure 2B). Correlation analysis between TE values and translation or transcript levels across the first ten clusters showed that TE correlated positively with translation levels from Phases I to IV, but this relationship was reversed in Phase V (Figure S5).

To compare differences between phases at the transcriptional and translational levels, we conducted gene co‐expression and GO enrichment analyses for both the transcriptome and translatome datasets (Figure S6). GO enrichment of genes showing phase‐specific transcription or translation indicated that these genes participate in different biological processes during the early phases but converge toward similar processes after meiosis (Figure S6). For instance, genes associated with “male meiosis II” were enriched only in the transcriptome during Phase I, before meiosis. In Phases I and II, GO enrichment terms differed substantially between the transcriptome and translatome, whereas in the later phases, particularly in pollen, enrichment patterns were largely consistent (Figure S6). These findings suggest that translational regulation contributes significantly to gene expression control across different phases of maize anther development.

### 2.3 Translational Regulation Functions in Maize Anther Development

We next examined the effects of translational regulation on global gene expression across the ten developmental stages. Differences in translational regulation among genes are reflected by variation in TE, defined as the ratio of ribosome footprints to mRNA abundance. Such differences are expected to increase the overall variation in expression levels (expression variation) in the translatome compared with the transcriptome (Figure 2C). We estimated that translational regulation increased expression variation by 11%–56% across the ten stages (Figure 2C). The influence of translational regulation was greatest during the 1.5‐mm anther stage of Phases II and IV (Figure 2C). The TE of genes was relatively low during meiosis, reaching its minimum at the 1.5‐mm stage, and was highest at Phase IV (Figure 2D). This result is consistent with our previous observation that genes enriched in Phase IV are primarily involved in the GO term “translation” (Figure 2B).

To identify genes with disproportionately high translational efficiency (TE > 2, indicating that translation levels were more than twice the transcript levels) at specific stages, we classified 2544 genes as high TE genes. Genes were included if their transcript or translation levels were ≥50 (FPKM for transcription or RPKM for translation) at any stage (Data S2). Among these, the first 489 genes listed exhibited particularly high TE, exceeding 5 in at least one phase (Data S2). For example, *Zm00001eb212800* (*MS28*) was transcribed at a relatively low level during Phase I, yet its TE exceeded 5 (Figure S7A). Loss of function of *MS28* results in pollen sterility [40]. Another key gene, *MS26*, was highly expressed during Phase II, with translation levels fourfold higher than transcript levels (Figure S7B). Mutations in *MS26* also cause male sterility [41]. Similarly, *DRP1*, a high TE gene in Phase II, is associated with male sterility (Figure S7C) [42]. In pollen, *RALF2* translation levels were threefold higher than transcript levels (Figure S7D), and mutation of *ralf2/3* led to pollen tube bursting [43].

In contrast, 939 genes were classified as low TE genes (translation levels less than half the transcript levels) (Data S3). A typical example is *Zm00001eb198610*, which encodes the anther‐specific protein *Ms44*. This gene displayed a very high transcript level, but a low translation level at 2.0‐mm stage (Figure S7E), and a single base‐pair substitution in *Ms44* causes male sterility [44]. Ninety‐one additional genes exhibited fluctuating TE, showing high TE at one stage but low TE at another (Data S4). We speculated that genes with high or low TE are likely to play important roles in anther development. In total, 34 sterility‐related genes were significantly enriched relative to the background (*p* = 10^−16.39^; Figure S8; Table 1).

**TABLE 1:** Genes related to male sterility belong to the high or low TE group.

Phase	Gene Name	Gene ID	High or low TE	Anther Cytology
I	*MAC1*	Zm00001eb408950	High	Failed specification of somatic wall layers; excess AR proliferation [45, 46, 47]
I	*OCL4*	Zm00001eb024680	High	Extra subepidermal cell layer [48, 49]
I	*MSCA1*	Zm00001eb300050	High	No AR and somatic cells; parenchymal cells and vascular strands are formed [50, 51]
I	*MS28*	Zm00001eb212800	High	Premature vacuolization tapetum; sticky Ubisch bodies; severely wizened pollen grains [40]
I	*MS44*	Zm00001eb198610	Low	No dehiscent anthers [44]
I	*TGA9‐1* ^a)^	Zm00001eb197040	High	Smooth inner surface without Ubisch bodies and fewer knitting cuticle on the outer surface of anther wall [52]
I	*TGA10*	Zm00001eb316720	High	Anthers failed to dehisce [52]
II	*MS20*	Zm00001eb021500	High	Smaller and wilted anther; glossy and smooth anther outer face and inner face, no Ubisch body [53]
II	*MS23*	Zm00001eb332170	High	Extra tapetum periclinal divisions [54, 55, 56]
II	*MS26*	Zm00001eb005300	High	Spikelets with no evidence of emerging anthers [41]
II	*DRP1*	Zm00001eb267820	High	abnormal Ubisch bodies, defective tectum of the pollen exine, and complete male sterility [42]
II	*MS45*	Zm00001eb398090	High	Abortion of microspore development [57, 58]
II	*DCL5*	Zm00001eb045380	High	Short anthers with defective tapetal cells; temperature‐sensitive male fertility [59]
II	*ZmCOI2a* ^a)^	Zm00001eb147720	Higher	*coi2a coi2b* double mutant has non‐dehiscent anthers, delayed anther development and male sterile [60]
II	*ZmDFR2* ^a)^	Zm00001eb317040	High	Double homozygous mutant (*dfr1/2*) exhibits complete male sterility with smaller anthers and without visible pollen grains [61]
II	*ZmACOS5‐1* ^a)^	Zm00001eb083110	High	Double homozygous *zmacos5‐1/‐2* display complete male sterility without pollen grains [61]
II	*ZmACOS5‐2* ^a)^	Zm00001eb119040	High	Double homozygous *zmacos5‐1/‐2* display complete male sterility without pollen grains [61]
II	*MYB84*	Zm00001eb424660	High	Smaller anthers and no pollen grains [52, 62]
II	*ABCG26*; *MS2*	Zm00001eb386540	High	Completely collapsed microspores; complete male sterility [63, 64]
II	*IPE1*	Zm00001eb021500	High	Glossy outer anther surface; abnormal Ubisch bodies; and defective pollen exine [65]
II	*ZmPKSB*	Zm00001eb305800	High	Denser anther cuticle but thinner pollen exine; delayed tapetal degeneration [66]
II	*ZmTKPR1‐1* ^a)^	Zm00001eb035410	Low	*tkpr1‐1/‐2* exhibits collapsed microspore and completely male sterile [62]
II	*ZmTKPR1‐2* ^a)^	Zm00001eb317040	High	*tkpr1‐1/‐2* exhibits collapsed microspore and completely male sterile [62]
IV	*RALF3* ^a)^	Zm00001eb120660	Low	Double homozygous *ralf2/3* display altered cell wall organization and thickness culminating in pollen tube burst [43]
V	*RALF2* ^a)^	Zm00001eb338050	High	Double homozygous *ralf2/3* display altered cell wall organization and thickness culminating in pollen tube burst [43]
I & II	*INVAN6*	Zm00001eb230860	High	Meiotic arrest and significantly decreased pollen fertility [67]
I & II	*ZmMs13*	Zm00001eb221130	High	Premature tapetal programmed cell death; defective pollen exine and anther cuticle formation; complete male sterility [68]
II‐III	*ZmMs25*	Zm00001eb402470	High	Defective anther cuticles, abnormal Ubisch body, impaired pollen exine; complete male sterility [69]
I‐III	*MSP1*	Zm00001eb143420	High	Failure of tapetum and middle layer cell specification [51, 70]
II‐IV	*bHLH51*	Zm00001eb208200	High	Smooth inner and outer surfaces of anther wall without knitting cuticle and Ubisch bodies; no visible pollen grains [52, 56]
III‐V	*LBD10* ^a)^	Zm00001eb051480	High	*lbd10/27* had a relatively larger proportion of aborted pollen grains [52]
IV & V	*ZmCOI2b* ^a)^	Zm00001eb397990	Low	*coi2a coi2b* double mutant has non‐dehiscent anthers, delayed anther development and male sterile [60]
I‐IV	*MYB33‐2* ^a)^	Zm00001eb150140	High	Smaller anthers; non‐visible pollen grains [52]
I‐IV	*NDL1*	Zm00001eb351270	High	Reduced or even unbranched tassel at high temperatures [71]

^a)^ Represents redundant genes which are related to male sterility.

**FIGURE 3:**
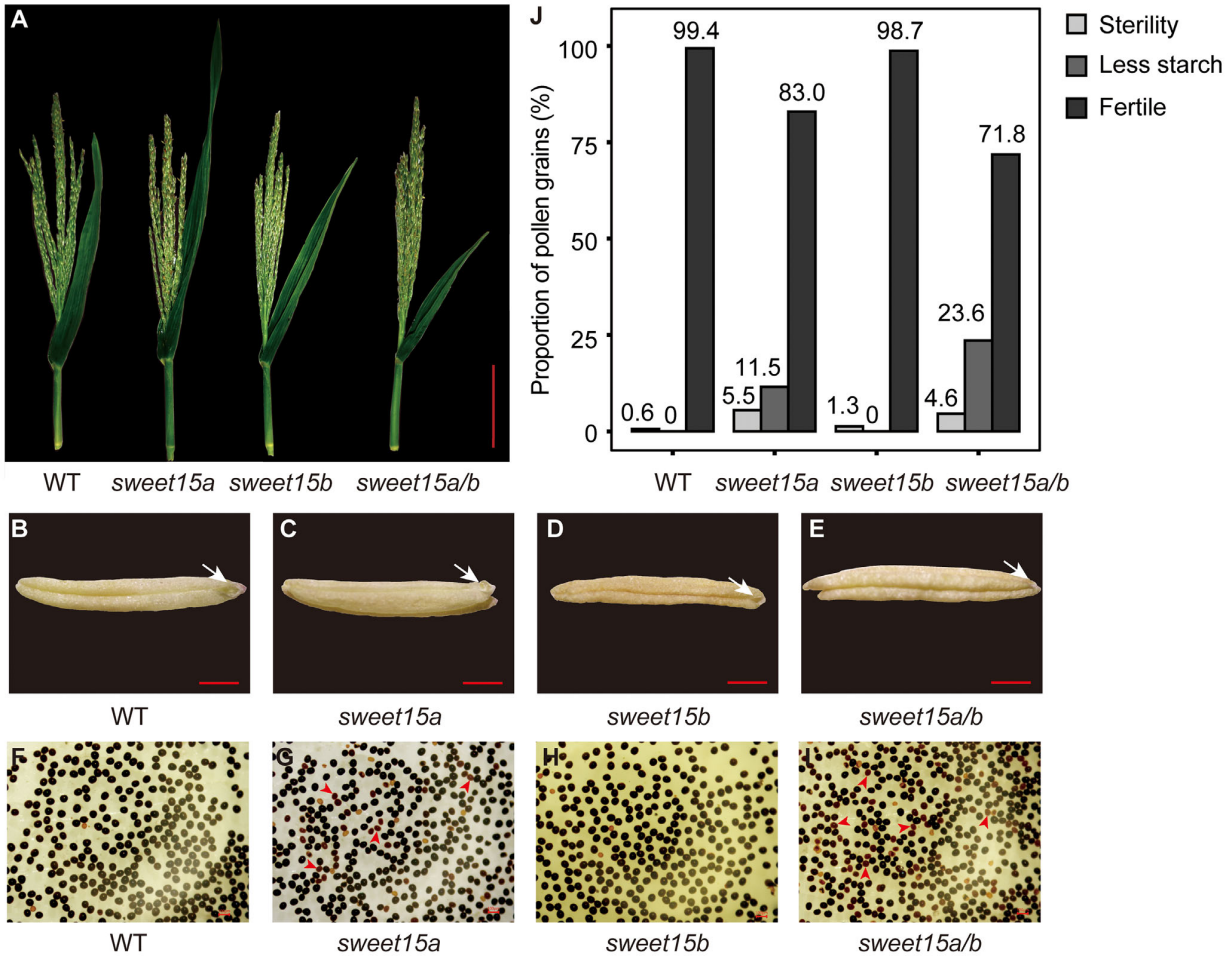
Tassel, anther, and pollen phenotypes of wild type (WT), *sweet15a*, *sweet15b*, and *sweet15a/b* mutants. (A) Tassel phenotype of WT and mutants. Scale bar, 10 cm. (B–E). Anther phenotype of WT and mutants. The white arrow indicates anther dehiscence. Scale bar, 1 mm. (F–I) Mature pollen stained with I_2_–KI. Red arrowheads mark pollen grains with reduced starch accumulation. Scale bar, 100 µm. (J) Quantification of mature pollen with different starch contents.

Genes associated with male sterility were predominantly enriched in Phases I and II. GO enrichment analysis indicated that these genes are mainly involved in protein degradation and carbohydrate metabolism (Figure S9). These findings suggest that genes related to biosynthesis and energy conversion during the later stages of anther development are crucial for normal fertility. For example, the *PME/PMEI* gene family participates in pectin synthesis in anthers and pollen and contributes to pollination and pollen tube elongation [72]. This family comprises 43 *PMEs* and 49 *PMEIs*, among which 24 showed high TE, and 5 showed low TE during anther development [73] (Data S2 and S3). Notably, *ZmPMEI1* is associated with pollen tube bursting [74]. Another study reported that 13 *PME/PMEI* genes were expressed exclusively in sterile anthers [73]. The *SWEET* gene family encodes sugar transporters that move sugars bidirectionally across membranes along concentration gradients [75]. In *Arabidopsis*, *AtSWEET5*, *AtSWEET8*, *AtSWEET13*, and *AtSWEET14* are essential for normal anther development [76, 77, 78]. We used MUSCLE (https://www.ebi.ac.uk/jdispatcher/msa/muscle?stype = protein) to align maize SWEET protein sequences with *Arabidopsis* SWEET proteins related to anther development (Figure S10A). We hypothesized that *ZmSWEET6a/b* and *ZmSWEET15a/b*, which exhibit high TE in anthers, may play key roles in anther development (Figure S10B). *ZmSWEET15a/b* showed particularly high TE during Phase II. Functional analysis demonstrated that the *sweet15a/b* double mutant produced more mature pollen grains with incomplete starch filling than the wild type or single mutants, while plant phenotype, tassel growth and anther dehiscence were unaffected (Figure 3) [79].

Translational regulation also differentially affects the expression of functionally redundant genes associated with male sterility. For example, the *tga9‐1/2/3* triple mutant, but not the single or double mutants, exhibited male sterility. Among these, only *TGA9‐1* showed markedly high TE during Phase I (Figure S11A–C). Likewise, *tkpr1‐1/‐2* double mutants, but not single mutants, displayed complete male sterility. The sterility phenotype of *tkpr1‐1/‐2* resulted from defects in the encoded proteins rather than reduced transcription. Translational regulation increased the TE of *ZmTKPR1‐1* while decreasing that of *ZmTKPR1‐2*, even though both genes are transcriptionally activated by *ZmMYB84* and are functionally redundant (Figure S11D,E) [62]. These findings indicate that translational regulation contributes to anther development by modulating the expression of functionally redundant genes. In summary, differences in TE, whether high or low, play crucial roles in anther development and may underlie distinct expression patterns among redundant genes.

### 2.4 Maize Anthers Carry Out Photosynthesis With Lower Efficiency

**FIGURE 4:**
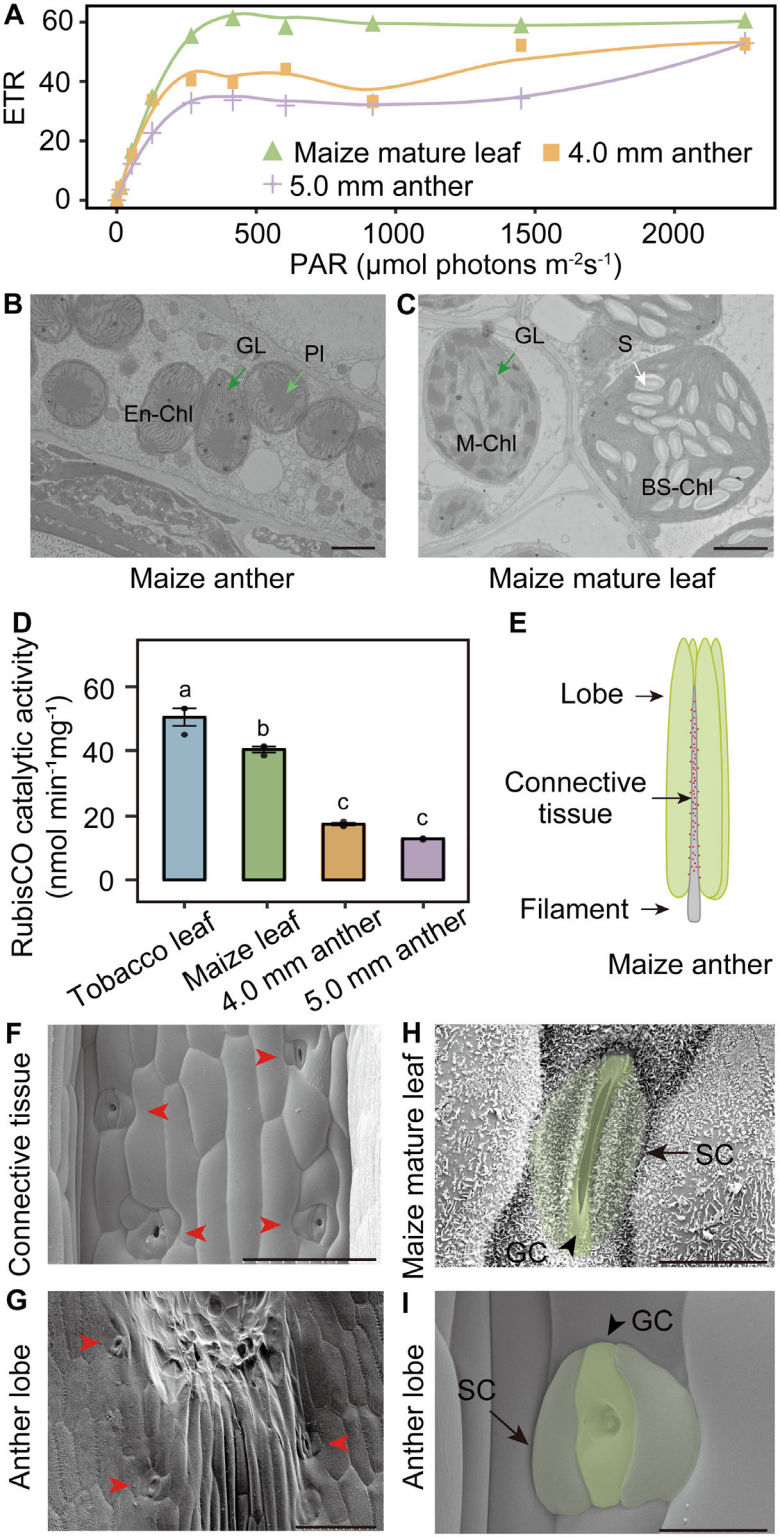
Maize anthers exhibit weak photosynthetic activity. (A) Photosynthetic electron transport rate (ETR) in mature maize leaves and 4.0‐ and 5.0‐mm anthers. (B,C) Chloroplast ultrastructure in the endothecium of a maize anther (B) and in mature leaf mesophyll cells (C). En‐Chl, chloroplasts in the endothecium of a maize anther; M‐Chl, chloroplasts in mesophyll cells of a maize leaf; BS‐Chl, chloroplasts in bundle sheath cells of a maize leaf; PI, prolamellar body; GL, grana lamella; S, starch. Scale bar, 2 µm. (D) Catalytic activities of ribulose‐1,5‐bisphosphate carboxylase/oxygenase (Rubisco) in tobacco leaf, maize leaf, and 4.0‐ and 5.0‐mm anthers. Tobacco leaf was used as the C_3_ photosynthesis control. Each sample represents three biological replicates. Statistical differences were assessed using a *t*‐test, *n* = 3. (E) Schematic representation of stomatal distribution on maize anthers. Red dots indicate the locations of anther stomata. (F) and (G) Transmission electron microscopy (TEM) images showing stomata in maize anther connective tissue (F) and on the adaxial side of anther lobes (G). Red arrowheads indicate stomata. Scale bar, 10 µm. (H) and (I) Scanning electron microscopy (SEM) images showing stomata in mature maize leaf (H) and anther (I). SC, subsidiary cell; GC, dumbbell‐shaped guard cell. Scale bar, 2 µm.

GO enrichment analysis indicated that photosynthesis‐related genes were enriched in Phase III (Figure S6). Previous studies suggested that anthers begin photosynthesis at S10 [80]. Chlorophyll fluorescence showed that anthers have photosynthetic potential comparable to that of mature maize leaves after the 4.0‐mm stage (Phase III) (Figure S12). The electron transport rate (ETR) of anthers was lower than that of mature leaves, which may reflect the fact that chloroplasts in anthers contain fewer mature grana lamellae and more prolamellar bodies than leaf chloroplasts (Figure 4A–C). In addition, the catalytic activity of ribulose‐1,5‐bisphosphate carboxylase/oxygenase (Rubisco), a key rate‐limiting enzyme in photosynthesis, was lower in anthers than in leaves (Figure 4D). These results indicate that the photosynthetic efficiency of anthers is substantially lower. It remains unclear whether anthers, as reproductive organs, can assimilate CO_2_ in a manner similar to leaves. Interestingly, a small number of stomata were observed in the connective tissues and adjacent regions on the adaxial side of anthers, resembling the stomata on the epidermis of maize leaves (Figure 4E–I). Moreover, *Zm00001eb158800*, a member of the *β*‐carbonic anhydrase (*β*‐CA) family, was translated with high efficiency even though the C_4_‐type *β‐CA* gene was almost not expressed (Figure S13). Furthermore, to verify whether anthers can assimilate CO_2_, the micro‐gas exchange system was utilized to measure the net photosynthetic rate of anthers of maize. The data showed that the anthers assimilated CO_2_ in a very weak degree compared to leaves, and the net photosynthetic rate was negative (Figure S14). Together, these observations suggest that anthers can perform photosynthesis with low efficiency and assimilate CO_2_ via a pathway distinct from typical C_4_ photosynthesis.

### 2.5 Different Characteristics of 5' ORFs and 3' ORFs May Have Opposite Effects on mORFs Translation

Ribosome profiling enables the identification of translatable ORFs across the entire genome. Recent studies have shown that translatable ORFs in UTRs can influence the translation of their corresponding mORFs [25, 27]. To investigate whether this occurs during anther development, we identified 761, 175, and 268 translated ORFs located in 5′ UTRs, 3′ UTRs, and annotated noncoding regions, respectively. ORFs in UTRs were defined as 5′ ORFs, 3′ ORFs, or noncoding ORFs (ncORFs) when they shared a termination codon and their nonoverlapping sequences displayed three‐nucleotide periodicity (Figure S15 and Data S5). Analysis of Kozak sequence features surrounding the first AUG (with A or G at the upstream −3 position and G at the downstream +4 position) showed that these ORFs have strong potential for translation, similar to the mORFs of protein‐coding genes [81] (Figure S16A–D).

**FIGURE 5:**
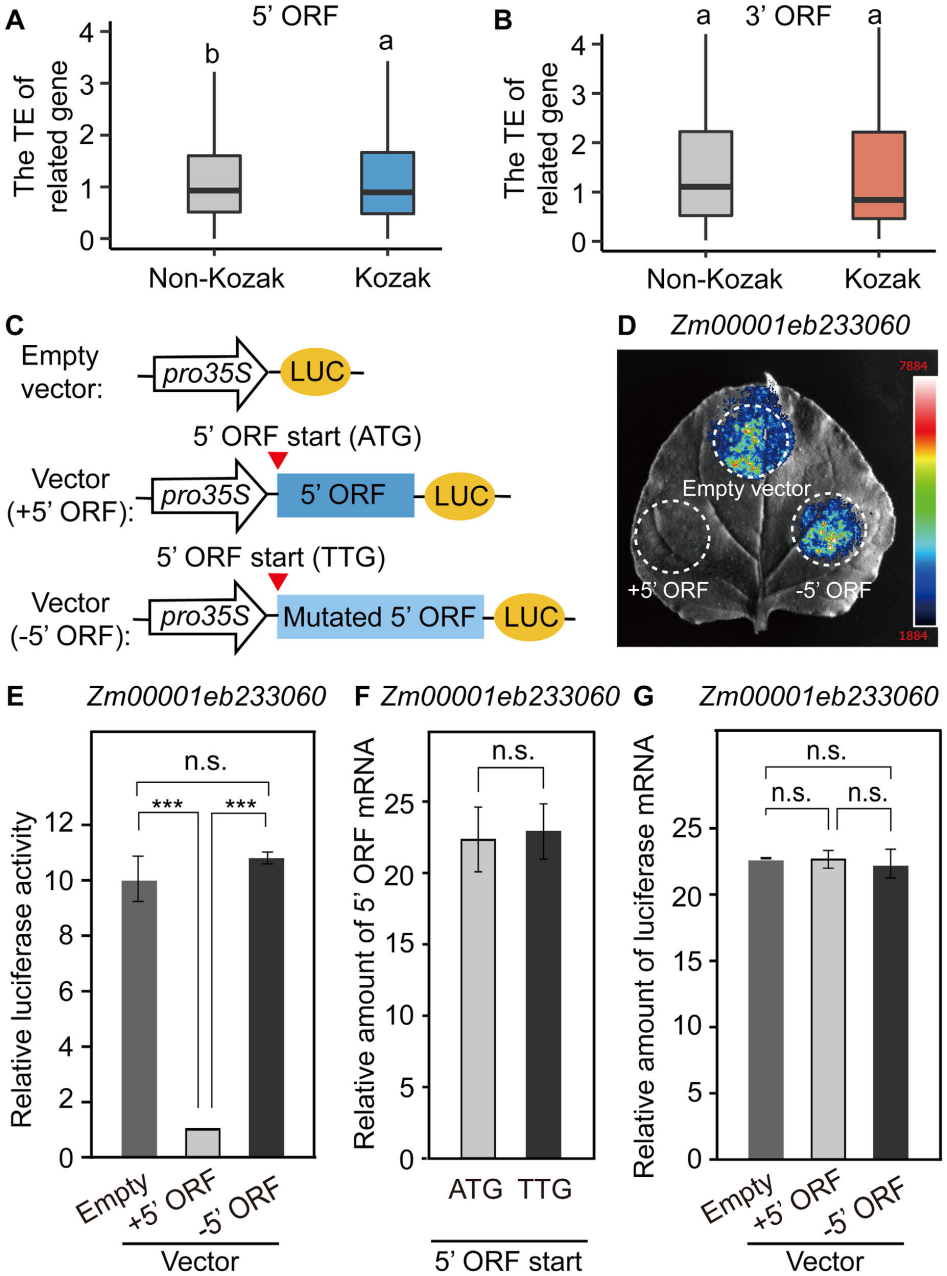
Adjacent isoforms may repress mORF translation. (A,B) Boxplots showing relationships between the presence of Kozak sequence characteristics in 5′ ORFs (A) or 3′ ORFs (B) and the translation efficiency (TE) of their corresponding genes. Different lowercase letters indicate significant differences (*p < 0.05) determined by Tukey’s honestly significant difference (HSD) test. (C) Schematic representation of luciferase reporter constructs: blank (*pro*35S: LUC), +5′ ORF fusion (*pro*35S:+5′ ORF: LUC), and −5′ ORF fusion (*pro*35S:−5′ ORF: LUC). *pro*35S, 35S promoter; LUC, firefly luciferase. +5′ ORF represents the original 5′ ORF, and −5′ ORF represents the start codon–mutated 5′ ORF (ATG → TTG). (D) Inhibitory effect of 5′ ORFs in the 5′ UTR on translation of the main ORF in the cognate gene. Images show LUC fluorescence in 4‐week‐old *Nicotiana benthamiana* leaves infiltrated with *Agrobacterium* suspensions containing the indicated vectors. The leaf tip was infiltrated with the empty vector, and the left and right sides were transformed with +5′ ORF and −5′ ORF constructs, respectively. White dashed circles indicate the injection areas used for fluorescence detection. (E) Relative luciferase activities of the empty, +5′ ORF, and −5′ ORF vectors corresponding to panel (D). n.s., not significant; ^***^ *p* <0.001. Statistical significance was assessed by a *t*‐test, *n* = 3. (F) The mRNA level of 5′ ORF and mutated 5′ ORF for *Zm00001eb233060*. n.s. indicates no significant difference. Statistical significance was assessed by a *t*‐test, *n* = 3. (G) The LUC mRNA levels of empty vectors, +5′ ORF vectors, and ‐5′ ORF vectors for *Zm00001eb233060*. n.s. indicates no significant difference. Statistical significance was assessed by a *t*‐test, *n* = 3.

The TE of genes containing 5′ ORFs with strong Kozak features was higher than that of genes lacking these features (Figure 5A). However, no significant difference was observed in the TE of genes with 3′ ORFs regardless of the presence of strong Kozak features (Figure 5B). Given the study in humans have shown that different characteristics of ORFs derived from UTRs of genes (such as the number and length of ORFs) have varying impacts on gene translation efficiency [82, 83], we analyzed the relationship between the TE of genes containing different numbers and lengths of 5' and 3' ORFs. The data showed that genes containing 5′ ORFs exhibited lower TE compared with those without 5' ORFs, and the number of 5' ORFs may be related to the lower TE (Figure S16E). Whereas genes containing 3′ ORFs showed the opposite trend, and this effect was not dependent on the number of 3′ ORFs (Figure S16F). Furthermore, the TE of genes was negatively correlated with the length of their 3′ ORFs, while the length of 5′ ORFs had no effect (Figure S16G,H). These findings suggest that 5′ and 3′ ORFs with different characteristics have opposite effects on gene translation.

Because the 5′ ORFs identified were located in the UTRs of their cognate genes, we hypothesized that they could inhibit translation of the mORFs, similar to uORFs [25, 27, 28]. However, we observed no strong negative correlation between the translation level of 5′ or 3′ ORFs and the TE of their corresponding genes (Figure S17), consistent with findings from human heart, where no anticorrelation between uORF and mORF translation was detected [25]. To test whether specific 5′ ORFs affect translation of their cognate mORFs, we selected three genes—*Zm00001eb376970*, *Zm00001eb291900*, and *Zm00001eb233060*—and inserted their 5′ ORFs into luciferase (LUC) reporter vectors (+5′ ORF constructs). These constructs were introduced into tobacco (*Nicotiana benthamiana* L.) leaves via *Agrobacterium tumefaciens*‐mediated transformation (Figure 5C,D; Figure S18A,B). Mutated constructs in which the start codon (ATG) was replaced with TTG (−5′ ORFs) served as negative controls alongside empty vectors. The LUC fluorescence intensity of +5′ ORF constructs was significantly lower than that of −5′ ORF and empty vectors (*p* < 0.001; Figure 5D,E; Figure S18A–C), indicating that 5′ ORFs in the UTRs of their respective genes inhibit translation of downstream mORFs. For each of the three genes tested, there were no significant differences in mRNA levels between the intact and mutated 5′ ORFs, nor among LUC mRNA levels for empty, +5′ ORF, and −5′ ORF constructs (Figure 5F,G; Figure S18D,E). These results indicate that the inhibitory effect of 5′ ORFs on downstream translation occurs at the translational rather than the transcriptional level.

### 2.6 Independent Isoforms From Translated 3′ ORFs Could Affect Anther Development

**FIGURE 6:**
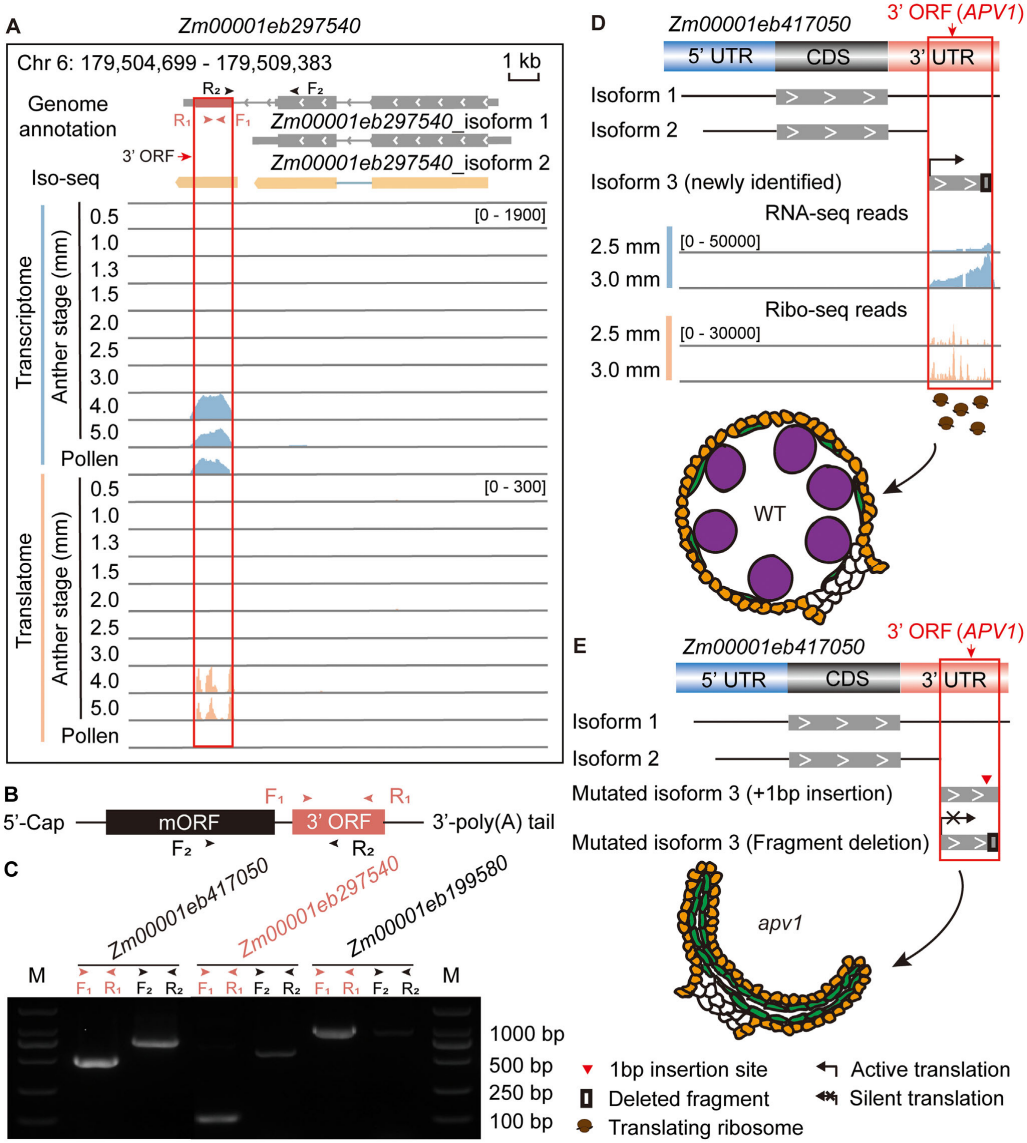
Independently transcribed and translated 3′ ORFs in maize anthers are associated with male sterility. (A) The 3′ ORF of *Zm00001eb297540* was dominantly expressed during the 4.0–5.0‐mm stages of maize anthers and in pollen as an isoform defined by full‐length transcriptome (Iso‐seq) analysis. The red box circled region denotes the 3′ ORF of *Zm00001eb297540*, which location is Chr 6: 179,505,444‐179,505,860. (B) Schematic representation of independent amplification of the 3′ ORF. Vermilion boxes indicate the translated 3′ ORF identified by ribosome profiling. (C) Agarose gel electrophoresis showing that 3′ ORFs from five genes can be independently transcribed in maize anthers. The 3′ ORF of *Zm00001eb417050* was amplified using the cDNA library of 3.0‐mm anthers, whereas the 3′ ORFs of *Zm00001eb297540* and *Zm00001eb199580* were amplified using the cDNA library of 4.0‐mm anthers. M, DNA marker (BM401‐02, TRANS, China). (D) Newly identified isoform of *Zm00001eb417050*, indicated by 3′ ORF (*APV1*), translated during the 2.5‐ and 3.0‐mm stages and associated with the fertile phenotype of maize anthers. (E) A large‐segment deletion of 65 amino acids or a frameshift mutation caused by a single‐base insertion in the peptide encoded by *Zm00001eb417050_*3′ ORF (*APV1*) resulted in the *apv1* phenotype, characterized by anther shrinkage and microspore sterility (Figure S21 and S22). Vermilion arrowheads indicate primers in the 3′ ORF region, and black arrowheads indicate primers used to detect the 3′ ORF–mORF fusion region. F, forward primer; R, reverse primer. In panels (A) and (B), vermilion boxes represent translated 3′ ORFs identified by ribosome profiling. The genome annotation for *Zm00001eb297540* was obtained from MaizeGDB (https://www.maizegdb.org/). Yellow boxes, or yellow boxes connected by light‐blue lines in the Iso‐seq panel, indicate identified isoforms of *Zm00001eb297540*. In panels (D) and (E), areas circled by a red box represent the 3′ ORF of *Zm00001eb417050*, which are matched to the cloned APV1 locus with the location of Chr 10: 86,322,132‐86,323,736, indicated by 3′ ORF (*APV1*). Numbers in the read‐coverage plots indicate maximum read‐depth ranges. Chr, chromosome; ORF, open reading frame; UTR, untranslated region; CDS, coding sequence.

We manually examined all identified ORFs by analyzing gene structural characteristics using full‐length transcriptome (Iso‐Seq) data. Fifty‐two of these ORFs corresponded precisely to newly identified isoforms of known genes (Data S6). One example is the 5′ ORF of *Zm00001ed260510*, which was detected to overlap with a separate isoform expressed across nine anther developmental stages (Figure S19A). Another new isoform derived from the 3′ ORF of *Zm00001eb297540* was expressed in 4.0‐ and 5.0‐mm anthers and in pollen (Figure 6A–C). In addition, the 5′ ORF from *Zm00001ed260510* and eight 3′ ORFs, including the 3′ ORF of *Zm00001eb297540*, which were newly defined to be transcribed from independent isoforms, were verified to be translated into peptides using MS data obtained from spikelets containing 1.5–2.0‐mm anthers (Phase II) (Data S6 and S7). In total, we identified 123 translated ORFs (Data S7). These findings suggest that some ORFs within UTRs may be translated independently of their cognate genes.

Interestingly, the translated 5′ ORF of *Zm00001eb233060*, which inhibits translation of its corresponding gene, was also transcribed as a newly identified isoform (Figure 5D,E; Figure S19B,C and Data S6). However, whether these independently translated ORFs from UTRs are functional in maize anther development remains unclear. A BLASTP search of peptides encoded by 5′ and 3′ ORFs across the genome revealed that the peptide encoded by the 3′ ORF of *Zm00001eb417050* was expressed in anthers at the 2.5‐ and 3.0‐mm stages of Phase II at an exceptionally high level (Figure S20). This peptide matched the Iso‐Seq–detected isoform and shared 94% sequence similarity with APV1, a protein reported by Somaratne et al. (2017) that contains a domain of the cytochrome P450 superfamily (Figure S21) [84]. Both the 65‐amino acid deletion mutant of *APV1* (corresponding to the 3′ ORF) and a frameshift mutation caused by a single‐base insertion resulted in defective pollen maturation and male sterility (Figure 6D,E; Figures S21 and S22) [84]. Together, these findings indicate that functional isoforms encoded by 3′ ORFs within UTRs may play important roles in maize anther development.

## 3 Discussion

Studies in multiple species have shown dynamic changes in gene translation during organ development [19, 20, 22]. Transcriptome studies have confirmed that changes in gene transcription are essential for maize anther development [6, 8, 9, 11]. Remarkably, however, the maize anther translatome has remained largely uncharacterized.

Here, we analyzed transcriptome and translatome data from ten key stages of maize anther development and observed substantial differences in gene transcription and translation across developmental phases, except for the pollen phase (Figure 1A; Figure S6). Previous transcriptome studies of early maize anther development reported that meiotic genes are expressed before meiosis [8, 10, 11]. Our results further revealed that genes involved in biosynthesis at a given stage show enhanced TE during the preceding developmental stage (Figure 2B). For example, genes involved in sporopollenin and very long‐chain fatty acid biosynthesis were specifically enriched in Phase II, the meiotic phase (Figure 2B). This may indicate that the anther prepares the necessary genetic material in advance before entering important developmental processes, which may be controlled by translational regulation. In addition, we observed a high correlation between TE and translation level from Phases I to IV but a lower correlation in Phase V (Figure S5), suggesting that translational regulation operates dynamically at different stages of anther and pollen development. This may be attributed to the distinct structures of pollen and anther, where pollen is an independent reproductive cell and lacks somatic cell layers. This provides insights for the subsequent studies on gamete development and somatic cell development, respectively.

Meiosis is a key stage in maize anther development and involves pronounced changes in transcriptional activity [2, 3, 8]. TE was lowest in Phase II, particularly at the 1.5‐mm stage (Figure 2D). We speculated that the completion of germ cell meiosis is of utmost importance, given that translation is energetically costly. Studies showed genes in anthers may be transcriptionally quiescent during meiosis, and the isolated meiotic cells had very similar expression profiles with the whole anther [2, 85]. Consequently, translation of genes might also be quiescent. After the anther completes mitosis at P IV, adjustments in biological process led to the enrichment of genes related to “translation”, which is similar to the dynamic regulation of transcription and translation during seed germination (Figure 2B) [86]. Enhanced translation during late mitosis likely supports the synthesis of sugars, lipids, and other metabolites necessary for pollen maturation [87, 88]. These observations explain the differing translation dynamics between the early (Phases I and II) and late (Phases III and IV) stages of anther development. Furthermore, many male‐sterility genes expressed in Phases I and II displayed high TE (Table 1). However, the contribution of biosynthesis‐related genes to fertility during late anther development remains poorly understood. To address this, we investigated the phenotype of the *sweet15a/b* mutant, noting that the accumulation of starch in mature pollen grains is affected (Figure 3). The large membership of the SWEET family (comprising 24 SWEETs, according to genome annotation in MaizeGDB; https://www.maizegdb.org/) suggests that the starch accumulation process in anthers may be regulated by multiple SWEETs. We hypothesized that the prior expression of *ZmSWEET6a* with high TE during the early development stage, along with the moderate expressions of *ZmSWEET1a* and *ZmSWEET11a*, may diminish the influence of *ZmSWEET15a/b* on anther development. A recent study also suggests the role of rice SWEET family genes in influencing starch accumulation in pollen, thereby affecting pollen fertility [89]. However, the current research on the role of non‐structural sugars in plant male reproductive organs remains poorly understood. In addition, we identified several genes with high TE values that are enriched in carbohydrate metabolic processes, emphasizing the importance of studying late‐stage biosynthetic events in anther development (Figure S9).

Previous work has demonstrated that maize anthers have photosynthetic potential [80, 90]. Although anthers and mature leaves exhibit comparable photosynthetic potential, the actual photosynthetic efficiency of anthers is much lower (Figure 4A,D; Figure S12). This may result from the prolonged darkness in which anthers develop, leading to chloroplasts with abundant prolamellar bodies and fewer mature grana lamellae (Figure 4B,C). Further evidence showed a negative net photosynthetic rate of maize anthers (Figure S14). The potential reasons could be that the photosynthetic capacity of the flower was rarely positive, and anthers, being a small component of the male reproductive organ, exhibit strong respiration [91, 92, 93, 94]. Their contribution to the photosynthesis of the whole flower is minor and may even be outweighed by the green palea and lemma of the spikelet [95]. Moreover, we observed that stomata with open structures were already visible at early stages of anther development (Figure S23). In the *msca1* mutant, stomata appeared on the surface of the anther, whereas the WT anther lacked them, which is consistent with our observations [50]. This is likely due to ectopic differentiation of parenchyma cells during early development, suggesting that cell fate determination has already occurred during anther primordium differentiation, indicating the formation of stomata. It is widely acknowledged that plants lack reproductive cell lines, and their reproductive organs, like vegetative organs (such as leaves), are differentiated from the shoot apical meristem. The reasons for retaining stomatal structures in anthers during differentiation, similar to those in leaves, and their evolutionary significance require further in‐depth research. Collectively, our findings suggest that anthers may assimilate CO_2_ via a pathway distinct from the typical C_4_ type. Further investigation is required to elucidate this mechanism.

This study verified the existence of peptides translated from 46 5' or 3' ORFs in anthers using MS and demonstrated that 5' ORF and uORF potentially share similar functions, specifically inhibiting the translation of downstream mORF (Figure 5C–E; Figure S18A–C). Numerous uORFs capable of inhibiting gene translation have been identified in humans and mammals, and many of these uORFs are associated with human diseases [24, 96]. Studies in plants have also shown that the inhibition of uORF on the translation of functional genes can affect the growth and development of plants. For instance, the inhibition of uORF upstream of *AtHB1* and *AtARFs* can affect the morphogenesis of Arabidopsis [97]. These studies implied that appropriate editing of uORF can alter the translation level of the target gene [26]. A recent study mutated the uORF of rice *OsABA2*, regulating its expression and thereby inhibiting the degree of preharvest sprouting, suggesting that utilizing uORFs may contribute to crop improvement [86, 98]. However, research on the relationship between the 5' UTR‐derived ORFs and functional genes in maize is still scarce. Future research could focus on the functions of some 5' ORF‐containing genes during anther development, given the availability of data resources from translatome analysis.

Gene isoforms in vertebrates play key roles in organ development; for instance, different versican isoforms contribute to distinct aspects of tooth development in mice [99], and *PARK2* isoforms display region‐specific distribution in the rat brain [100]. Among our findings, a notable isoform was derived from the 3′ UTR of *Zm00001eb417050*, identified as *APV1*, which is expressed in the tapetum and microspores and validated by full‐length transcriptome (Figure S20 and Data S6). Mutation of this isoform causes male sterility in maize [84] (Figure 6D,E; Figures S21 and S22). This result suggests that translatable ORFs derived from UTRs can influence anther development. The physiological relevance of these newly identified isoforms in specific developmental stages and cell types remains to be determined. Translated ORFs within 3′ UTRs are widespread in diverse species, including wheat, mouse, and human, but their potential translation as independent isoforms and their developmental roles are rarely examined [19, 25, 33, 36]. Our findings provide a framework for future studies on isoforms derived from UTRs and a theoretical basis for utilizing 5′ and 3′ ORFs to generate male‐sterile mutants in maize. Moreover, specific inhibition, weakening, or enhancement of the translation of these peptides will be crucial for elucidating the specific contributions of these noncanonical peptides to pollen fertility.

Previous research reported that *Zm401*, expressed in maize anthers as either a short ORF (sORF)–containing mRNA encoding an 89–amino acid protein or a noncoding RNA (ncRNA), regulates tapetum and microspore development [101]. Another study identified a translatable ORF, *qKDR1*, located in an intergenic region approximately 10 kb upstream of *RPG* (Regulated Peptide Gene), which influences kernel dehydration [102]. These examples highlight the regulatory potential of coding ORFs originating from intergenic regions. In the present study, we identified 268 translated ncORFs in intergenic regions. To date, the functions of peptides derived from ncORFs in maize anthers remain unexplored. Overall, our results underscore the importance of studying the translation of ORFs from both untranslated and intergenic regions to fully understand their roles in maize anther development.

## 4 Materials and Methods

### 4.1 Whole‐Transcriptome and Translatome Data Alignment

Ribo‐seq and RNA‐seq data from 0.5‐, 1.0‐, 1.3‐, 1.5‐, and 2.0‐mm anthers of Chang7‐2 were downloaded from NCBI under accession number PRJNA1029358. Data from 2.5‐, 3.0‐, 4.0‐, and 5.0‐mm anthers and from mature pollen grains were downloaded under accession number PRJNA1029386.

Low‐quality reads and sequencing adapters were removed using Skewer v0.2.2 [103]. For RNA‐seq, the parameters were “‐x AGATCGGAAGAGCACACGTCTGAACTCCAG TCA ‐y AGATCGGAAGAGCGTCGTGTAGGGAAAGAGTGT ‐Q 25 ‐l 35,” and for Ribo‐seq, the parameters were “‐x AGATCGGAAGAGCACACGTCTGAACTCCAGTCAC ‐Q 25 ‐l 25 ‐L 40.” Reference sequences for rRNA, tRNA, snoRNA, and snRNA were extracted from the maize genome (B73_RefGen_v5, https://ftp.ensemblgenomes.ebi.ac.uk/pub/plants/release‐62/gff3/zea_mays/). Reads were aligned to these reference sequences using Bowtie v1.3.0 [104] with the parameter “–un” to filter out reads derived from rRNA, tRNA, snoRNA, and snRNA. Cleaned RNA‐seq reads were aligned to the maize genome (B73_RefGen_v5, https://ftp.ensemblgenomes.ebi.ac.uk/pub/plants/release‐62/gff3/zea_mays/) using HISAT2 v2.2.0 [105] with the parameters “–rna‐strandness RF –dta‐cufflinks.” Cleaned Ribo‐seq reads were aligned to the maize genome (B73_RefGen_v5) using STAR v2.7.7a with the parameters ″–outFilterType BySJout –alignIntronMax 200000 –outSAMtype BAM SortedByCoordinate –quantMode TranscriptomeSAM GeneCounts –outFilterMismatchNmax 2 –outFilterMultimapNmax 1 –alignEndsType EndToEnd. Mapped reads were sorted and merged using SAMtools v1.9 [106].

### 4.2 Calculation of Transcript and Translational Abundance

Raw read counts were obtained using featureCounts v2.0.0 [107] with the parameters “‐O ‐p ‐s 2 ‐t exon ‐g gene_id” for RNA‐seq and “‐O ‐s 1 ‐t CDS ‐g gene_id” for Ribo‐seq. Gene expression levels were calculated as fragments per kilobase of exon per million mapped reads (FPKM) for RNA‐seq and reads per kilobase per million mapped reads (RPKM) for Ribo‐seq using the formula: FPKM or RPKM = (raw fragment count) / (gene exon or CDS length in kilobases) / (total mapped reads in millions). Translational efficiency (TE) was calculated as TE = (Ribo‐seq RPKM + 1) / (RNA‐seq FPKM + 1). Because the biological replicates were highly consistent (Figures S2 and S3), all replicates were processed using the same pipeline, and mean expression values were calculated for genes meeting the screening threshold of FPKM or RPKM ≥1 in at least one developmental stage.

### 4.3 Detecting 3‐nt Periodicity and Actively Translated ORFs

Noncoding RNAs (ncRNAs) from intergenic regions were identified using full‐length transcriptome data (accession number CRA025445 of the Big Sub, China National Centre for Bioinformation). Ribo‐seq footprints showing 3‐nt periodicity were calculated with RiboCode v1.2.12 [108] using the “metaplots” function with the parameter “‐f0_percent 0.6.” Actively translated ORFs were detected with RiboTaper v1.3 [109], and the file “translated_ORFs_filtered_sorted.bed” was used as the output for subsequent analyses. For ORFs sharing a termination codon but differing in length, the longer ORF was retained. Sequences near the start codons of different ORF types were extracted for Kozak sequence analysis, and these sequences were submitted to WebLogo3 (http://weblogo.threeplusone.com/) [110] to generate sequence motif plots. The relationships between UTR‐associated ORFs and translation efficiency (TE) of their corresponding mORFs were evaluated using Pearson correlations between ORF expression levels and gene TE across the ten developmental stages.

### 4.4 Chlorophyll Fluorescence Measurement

Fresh mature leaves and anthers were harvested immediately for measurement of the photosynthetic electron transport rate (ETR) and chlorophyll fluorescence (ChlF) using an IMAGING‐PAM chlorophyll fluorometer (Heinz Walz GmbH, Effeltrich, Germany).

### 4.5 Transmission Electron Microscopy and Scanning Electron Microscopy

To observe chloroplasts in maize anthers and leaves by transmission electron microscopy (TEM), fresh samples were collected and immediately fixed in 2.5% glutaraldehyde for ultrathin sectioning. Subsequent procedures were performed as described previously [42].

For scanning electron microscopy (SEM), fresh 5.0‐mm anthers and mature maize leaves were placed on plates and examined using a Hitachi S‐3400N scanning electron microscope (Hitachi, Tokyo, Japan).

### 4.6 Net Photosynthetic Rate Measurement

After placing the maize plants in a dark room for 30 min, fresh mature leaves and anthers at the 5.0‐mm stage were collected for measuring net photosynthetic rate. The net photosynthetic rate was measured by a Chlorophyll fluorescence‐imaging‐gas exchange simultaneous measurement system (GFS‐DUAL, WALZ, Germany).

### 4.7 Determination of Rubisco Catalytic Activity

Fresh tobacco (*Nicotiana benthamiana*) leaves, maize leaves, and 4.0‐ and 5.0‐mm anthers were collected and immediately frozen in liquid nitrogen. Approximately 0.1 g of ground frozen tissue from each sample was used to measure ribulose‐1,5‐bisphosphate carboxylase/oxygenase (Rubisco) catalytic activity with the Plant RCA Activity ELISA Kit (BR5400783, Bioleaper, China).

### 4.8 Evaluating the Degree of Regulation of Gene Expression

The degree of regulation of gene expression was represented by expression variance across the genome, where a higher variance indicates stronger regulation, as described by Wang et al. (2020) [35]. Genes from the transcriptome and translatome with median expression levels greater than 1 (FPKM for transcriptome or RPKM for translatome) in all ten stages were retained. Variance was calculated using the formula *S* ^2^_period_ = $\frac{1}{n}\;\sum_{i=1}^{n}\left(x_{i}-\;\bar{x}\right)$ ^2^ where *n* is the total number of genes, *i* represents an individual gene, and *x* represents its expression level.

### 4.9 Clustering and GO Enrichment Analysis

Clustering analysis was performed on genes from the ten developmental stages with minimum expression values greater than 1. The k‐means method in R was used with the parameters “cluster_num = 18, iter.max = 30, seed = 19960912.” For clustering analysis of expression differences among developmental stages, distances were calculated with the “dist” function in R using “method = euclidean,” followed by hierarchical clustering with “hclust” using “method = average.” Gene Ontology (GO) enrichment analysis was performed using the GO annotation file for maize genes. Significance testing was conducted in Python with a custom script employing the stats. hypergeom method.

### 4.10 Mass Spectrometry Database for 5′ and 3′ ORF Comparison

The maize inbred line Chang7‐2 was grown in a greenhouse under a 14‐h light (28°C) and 10‐h dark (22°C) photoperiod until the anther meiosis stage. Anthers measuring 1.5–2.0 mm were collected, immediately frozen in liquid nitrogen, and ground into powder. The mass spectrometry database of small peptides from meiotic anthers was prepared as described previously [111] for large‐scale identification of nonconventional peptides in maize and *Arabidopsis* through an integrated peptidogenomic pipeline. Briefly, the powdered anther tissue was heated in water at 95°C for 5 min, then precipitated in 10% (w/v) trichloroacetic acid/acetone solution at −20°C for 1 h. The precipitate was washed repeatedly with cold acetone until the supernatant was colorless, dissolved in 1% TFA solution containing plant protease inhibitors, and incubated at 4°C for 1 hour. The fractions were ultrasonicated on ice (40 W, 6 s per pulse, 8‐s intervals, repeated five times) and centrifuged at 10 000 × g for 20 min at 4°C. Supernatants were filtered through 10‐kDa molecular‐weight‐cutoff centrifugal filters (Millipore, MA, USA). Peptide mixtures were desalted using C18 cartridges (Empore SPE Cartridges C18, 7 mm inner diameter, 3 ml volume; Sigma), vacuum‐dried, and reconstituted in 40 µl of 0.1% TFA for LC–MS/MS analysis. The resulting peptidome dataset was deposited in ProteomeXchange under accession number PXD068789.

For peptide identification, raw MS data were converted to MGF format using Proteome Discoverer 1.4 (Thermo Fisher Scientific). Mascot (Matrix Science) was used to search against a customized database derived from ribosome sequencing. Mass tolerances for precursor and fragment ions were set to 5 ppm and 0.02 Da, respectively, and peptides with Mascot scores ≥20 were retained. Peptide fragments were matched to 5′‐ and 3′‐ORF protein sequences using the “re” library in Python, and matches indicated that peptides were derived from the respective ORFs.

### 4.11 RNA Extraction and RT‐PCR

Total RNA from 1.5‐, 2.0‐, 3.0‐, 4.0‐, and 5.0‐mm anthers was extracted using TRIzol reagent (Invitrogen, USA). Approximately 1 µg of total RNA was reverse‐transcribed to synthesize cDNA with the HiScript III 1st Strand cDNA Synthesis Kit (with gDNA wiper) according to the manufacturer's instructions (R312‐01, Vazyme, China). RT‐PCR was performed using the 2× Phanta Max Master Mix (Dye Plus) (P525‐02, Vazyme, China) in a 20‐µl reaction containing 10 µl of 2× Phanta Max Master Mix (Dye Plus), 1 µl of forward primer (10 mm), 1 µl of reverse primer (10 mm), 1 µl of cDNA, and 7 µl of nuclease‐free water. Primer sequences for all genes are listed in Data S8.

### 4.12 Vector Construction and Luciferase Assay

The coding sequence of each 5′ ORF under study was cloned into the pGreenII 0800+pActin2 plasmid (a modified version of pGreen‐0800‐LUC) to generate the proActin2:+5′ ORF‐LUC recombinant vector. A mutated version of the 5′ ORF, in which the start codon (ATG) was replaced with TTG, was cloned into the same plasmid to generate the proActin2:‐5′ ORF‐LUC vector. The constructs were introduced into the Agrobacterium tumefaciens strain GV3101. Bacterial cultures were grown in LB medium containing 50 µg ml^−^^1^ kanamycin (Sigma, USA) and 50 µg ml^−^^1^ rifampicin (Sigma, USA) at 28°C until OD_600_ = 0.8. Cells were collected by centrifugation at 5000 × g for 5 min at room temperature, resuspended in infiltration buffer (10 mm MES, pH 5.6; 10 mm MgCl_2_; and 100 µm acetylbutanone), and adjusted to a final OD_600_ of 0.5. The bacterial suspensions were infiltrated into Nicotiana benthamiana leaves using a 1‐ml disposable syringe. After incubation for 36 h at 25°C (day) and 22°C (night), infiltrated leaves were sprayed with 1 mm luciferin (dissolved in ddH_2_O containing 0.01% Triton X‐100). Fluorescence was visualized with a CCD camera (Tanon 5200), and relative luminescence intensity was quantified using ImageJ software. Co‐transformation of the empty vector and mutated (‐5′ ORF) vector into the same leaves served as negative controls. Luciferase fluorescence intensity was measured using the Dual Luciferase Reporter Gene Assay Kit (11402ES60, Yeasen Biotechnology, China). Relative LUC fluorescence was calculated by dividing the intensity of the +5′ ORF or −5′ ORF construct by the intensity of the blank vector. A circular region 1 cm in diameter (as shown in Figure 5D; Figure S18A,B) was harvested from each infiltrated site for analysis. Two leaves were used per replicate, and three biological replicates were included per treatment. Statistical significance was evaluated using a one‐tailed *t*‐test.

### 4.13 Statistical Analysis

All statistical analyses were performed in Python (SciPy package, version 1.14) and R (agricolae package, version 1.3). Adjusted *P*‐values for enrichment analyses were calculated using the hypergeometric distribution test, with significance thresholds set at *p* <0.05 and *p* <0.01. For multiple comparisons across groups, Tukey's honestly significant difference (HSD) test was applied, with distinct lowercase letters indicating statistically significant differences at *α* = 0.05. Error bars represent the standard error of the mean (SEM).

## Author Contributions

M.Z. and C.W. were responsible for conceptualization. Y.W., C.W., S.W., S.J., H.L., Y.H., and L.W. contributed to the methodology. M.Z., T.Z., and L.W. provided supervision. C.W. and M.Z. prepared the original draft of the manuscript.

## Funding

This research was supported by National Natural Science Foundation of China (32422063, U22A20474), National Key Research and Development Program of China (2022YFF1003502, 2022YFD1201802), Chengdu Institute of Biology, Chinese Academy of Sciences (QYJC2025‐1).

## Conflicts of Interest

The authors declare no conflicts of interest.

## Supporting information




**Supporting File 1**: advs74424‐sup‐0001‐SuppMat.docx.

**Supporting File 2**: advs74424‐sup‐0002‐DataFile.xlsx.

## Data Availability

All data are available in the main text or the supplementary materials.
