# Supplementary material for: Maize Anther Development Involves Translated Open Reading Frames From 3′ Untranslated Regions
Source: Adv Sci (Weinh). 2026 Feb 20;13(23):e23401. doi: 10.1002/advs.202523401 (PMC13104138; doi:10.1002/advs.202523401)
Supplement: Supplementary file 1 — Supporting File 1: advs74424‐sup‐0001‐SuppMat.docx. [file ADVS-13-e23401-s002.docx]

Supplementary Materials for

**Maize anther development involves translated open reading frames from 3′ untranslated regions**

Chunyu Wang *et al.*

Corresponding author: Liuji Wu, wuliuji@henau.edu.cn; Tao Zhang, zhangtao@cib.ac.cn; Mei Zhang, mei.zhang@ibcas.ac.cn

**This PDF file includes:**

Figs. S1 to S23

Legends for dataset 1

**Other Supplementary Material for this manuscript includes the following:**

Dataset 1


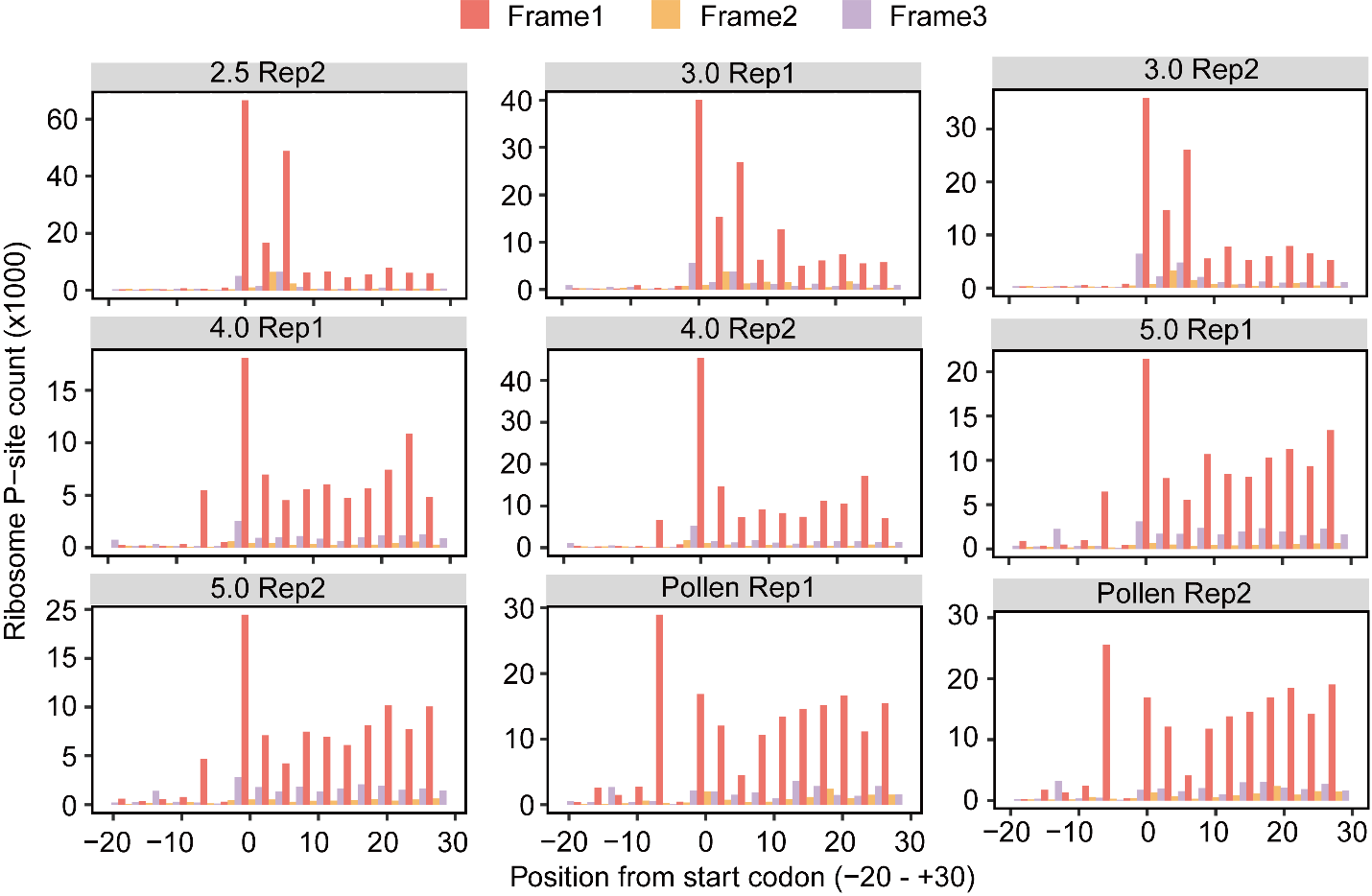


Figure S1. The 3nt periodicity of Ribosome profiling samples.

The bar charts display the P-sites of the Ribo-seq reads corrected for self-offset at the transcription start site -20 – +30 bp. Each replicate of four maize anther samples (2.5–5.0-mm) and mature pollen were shown, except the replicate 1 of 2.5-mm anther (Figure 1C).


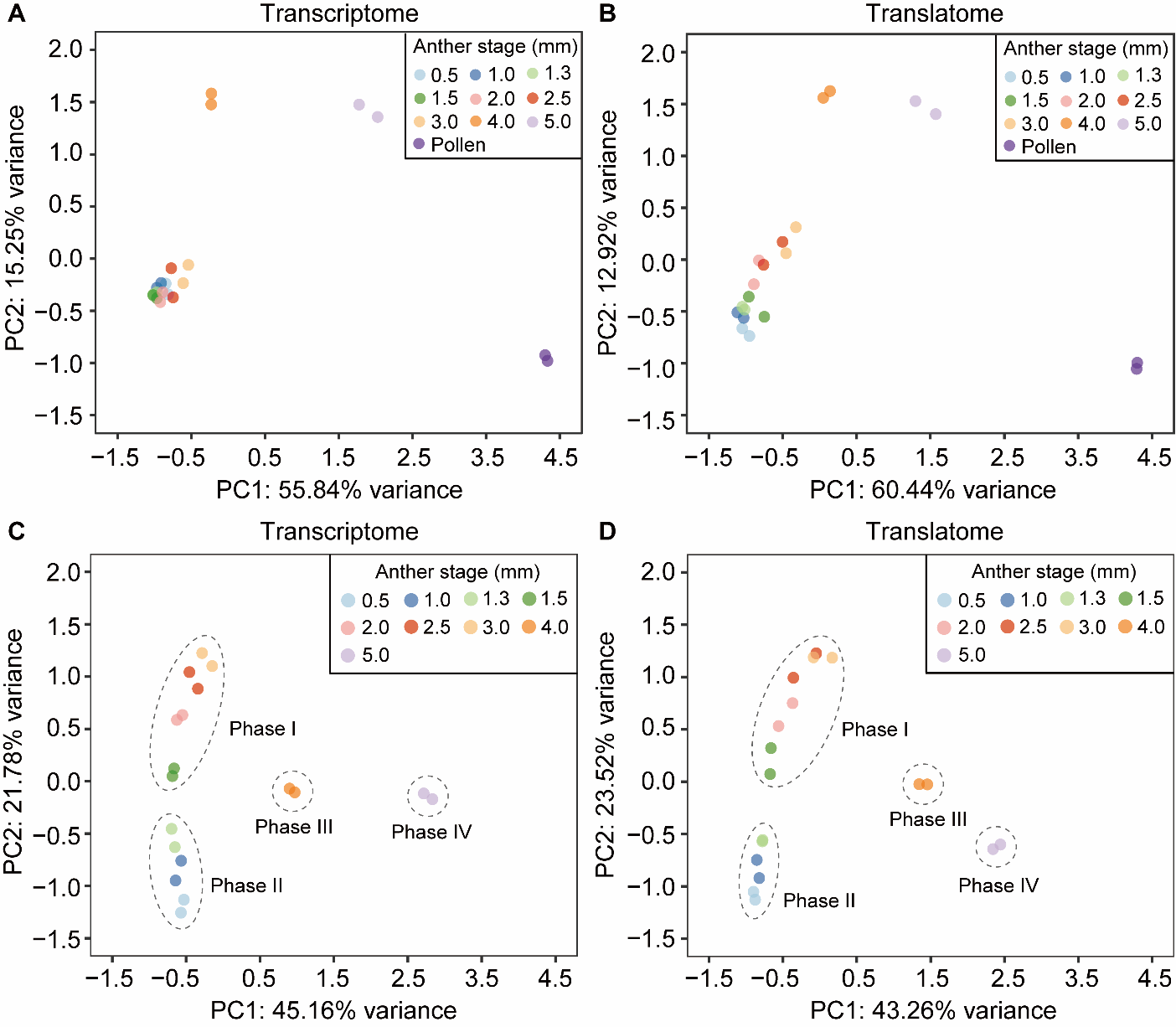


Figure S2. PCA analysis of transcriptome and translatome data from two replicates of maize anther samples.

A and B. PCA analysis utilized transcriptome and translatome data from two replicates of maize anthers across ten stages. The data revealed good reproducibility between two replicates at ten anther developmental stages.

C and D. PCA analysis utilized two sets of transcriptome and proteome data from anthers at nine different stages. The reason for excluding mature pollen is that it has a distinct structural difference from anthers, specifically the absence of a somatic layer. The classification of these stages by transcriptome and proteome was quite similar as shown in Figure 1A.


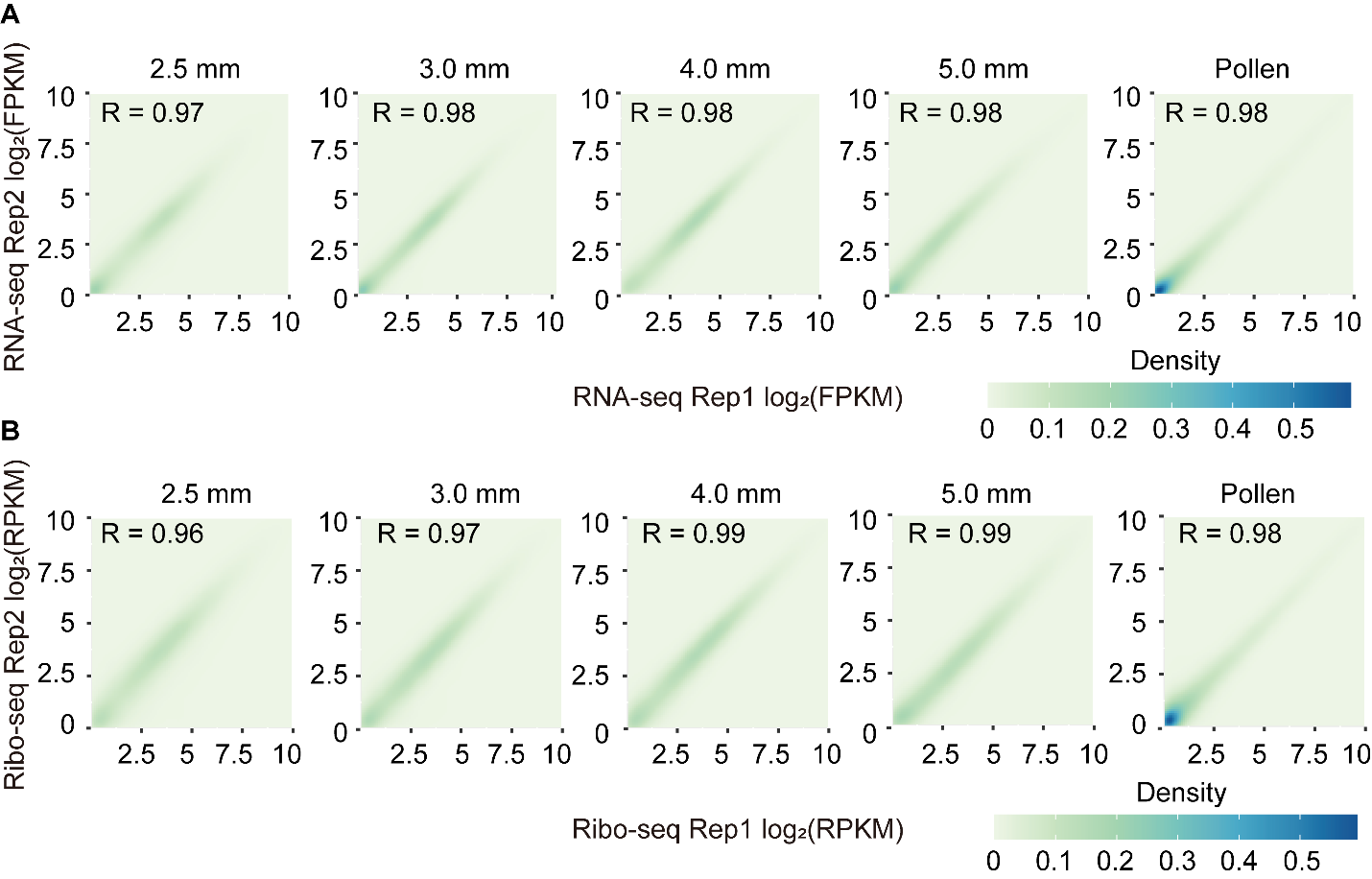


Figure S3. Correlation analysis of transcriptome data (A) and translatome data (B) at different developmental stages of maize anthers.

The calculation of gene transcription (FPKM, Fragments Per Kilobase of exon model per Million mapped fragments) and translation (RPKM, Reads Per Kilobase per Million mapped reads) levels are detailed in the Materials and Methods section. The correlation coefficient between every pair of replicates was then analyzed using log_2_(FPKM_RNA-seq_) or log_2_(RPKM_Ribo-seq_).


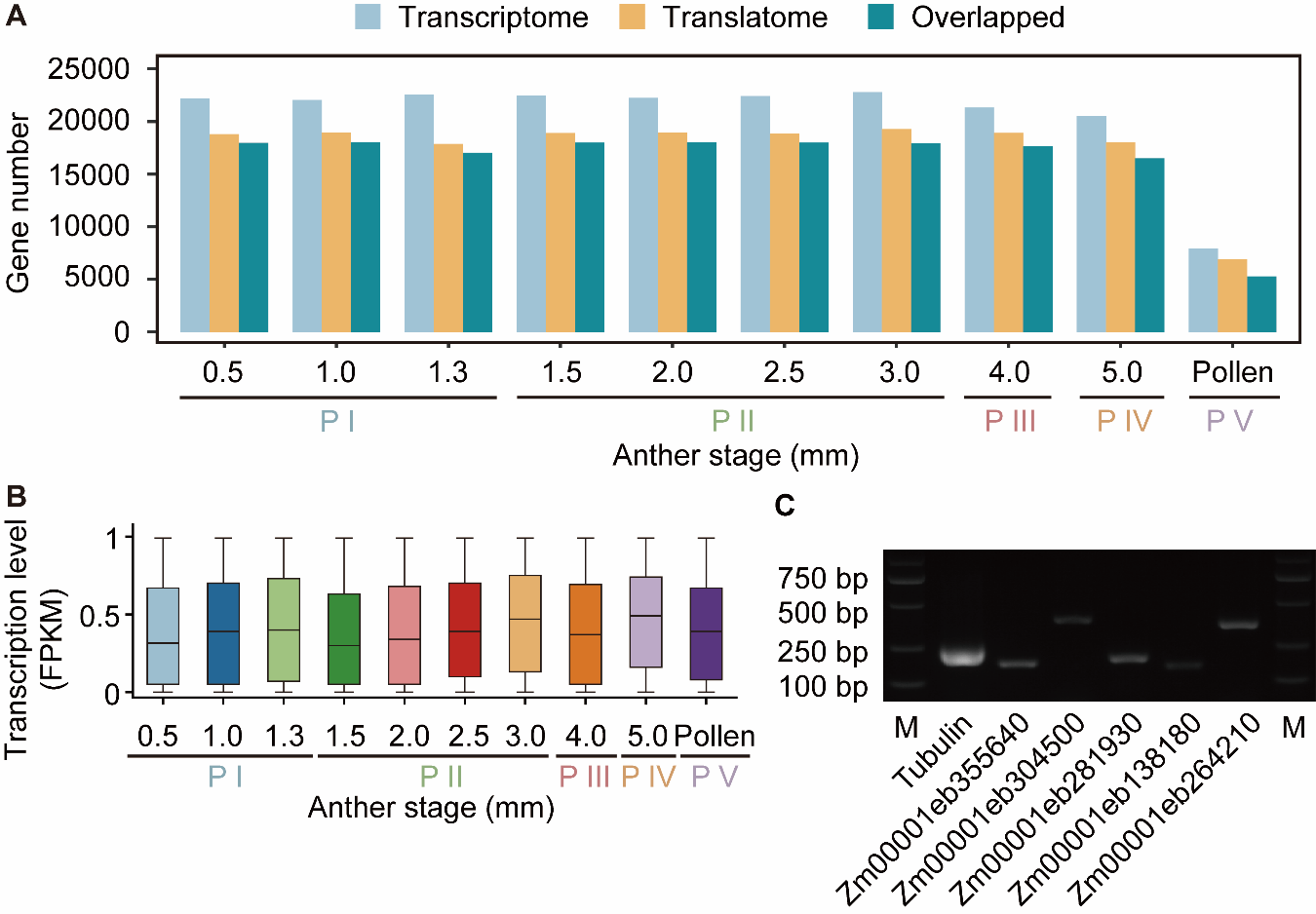


Figure S4. The genes filtered by threshold are transcribed in anthers.

A. Comparison of the number of genes transcribed and translated during ten developmental stages of maize anthers. The phase division of anther development is shown in Figure 1, A and E. The gene number represents the genes that identified as expressed genes in transcriptome (FPKM ≥ 1) or translatome (RPKM ≥ 1). Overlapped represents genes that are not only transcribed but also translated in specific stage. P, Phase. FPKM, Fragments Per Kilobase of exon model per Million mapped fragments. RPKM, Reads Per Kilobase per Million mapped reads.

B. The expression level of non-transcribed but defined as translated genes in ten stages.

C. Five randomly selected genes, defined as non-transcribed genes, were expressed in anthers. The housekeeping gene, *Tubulin*, was used as a control. M, DNA marker (BM401-02, TRANS, China).


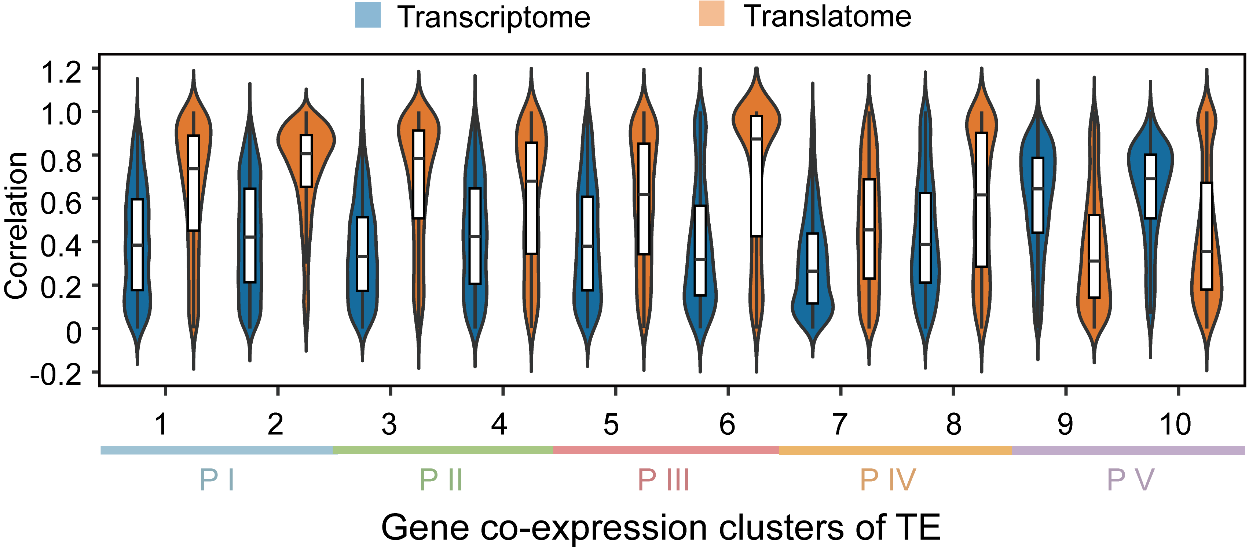


Figure S5. The correlation of the genes TE with transcriptome or translatome.

The violin plot represents the correlation between the level of gene transcription or translation and gene TE at different stages. The phase division of anther development is shown in Figure 1, A and E. The ten gene co-expression clusters are the first ten co-expression clusters of genes’ TE in Figure 2A. P, Phase. TE, translation efficiency.


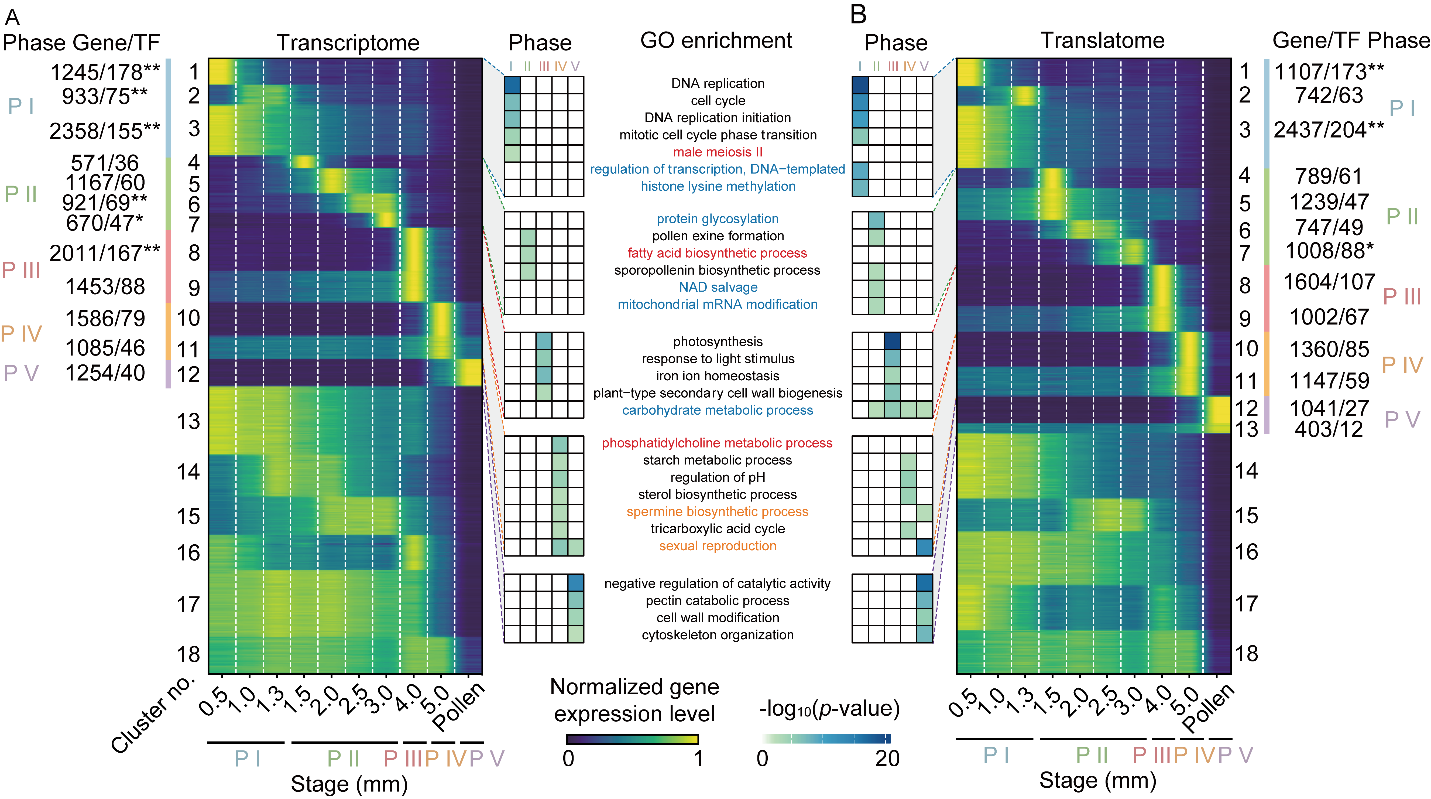


Figure S6. Gene co-expression in transcription level (A) and translation level (B).

The gene ontology (GO) enrichment analysis of the first 12 phase specific clusters of transcriptome (A) and the first 13 phase specific clusters of translatome (B) was shown in the middle lane. The numbers along the heatmap represent the gene and TF number of a certain cluster. The number to the left of the slash represents the number of genes, and the number of TF to the right. Asterisks represents significant enrichment of transcription factors in this cluster. During the same phase of GO enrichment, only transcriptional enriched terms were labeled in red, only translational enriched terms were labeled in blue, those enriched in both transcriptome and translatome were labeled in black, and transcription and translation are enriched in separate temporal phases were labeled in orange. The phase division of anther development is shown in Figure 1, A and E. TF, transcription factor. P, phase. No., number. Adjusted *p*-value was calculated by the Hypergeometric distribution test, **P* < 0.05, ***P* < 0.01.

**
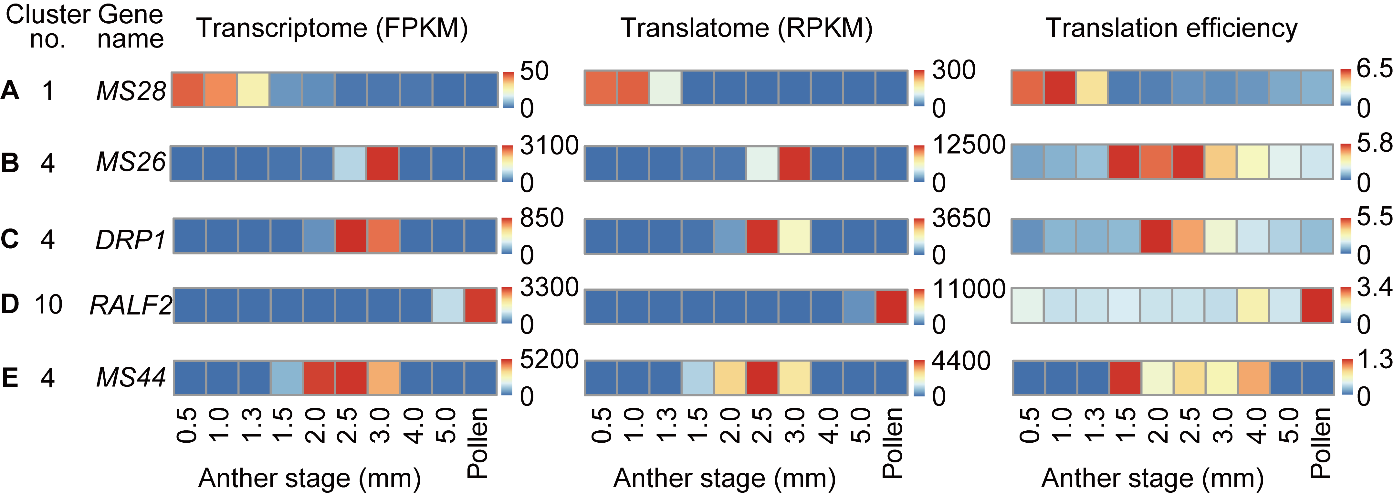
**

Figure S7. Transcript level, translation level and translation efficiency of 5 male sterility-related genes.

Each gene is shown with its corresponding cluster assignment, as defined in Figure 2A of the main text.

**
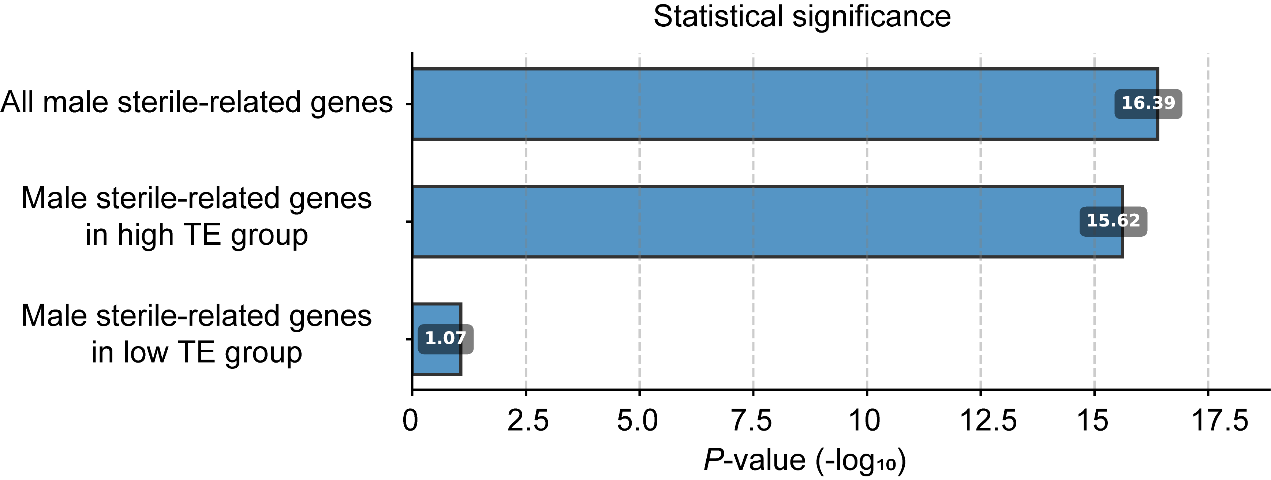
**

Figure S8. Statistical analysis of enrichment for sterility-related genes with high or low translation efficiency.

A total of 58 male sterility genes were included in the statistical analysis, with 30 classified as high TE genes and 4 as low TE genes. Statistical significance was assessed by Fisher's Exact Test.


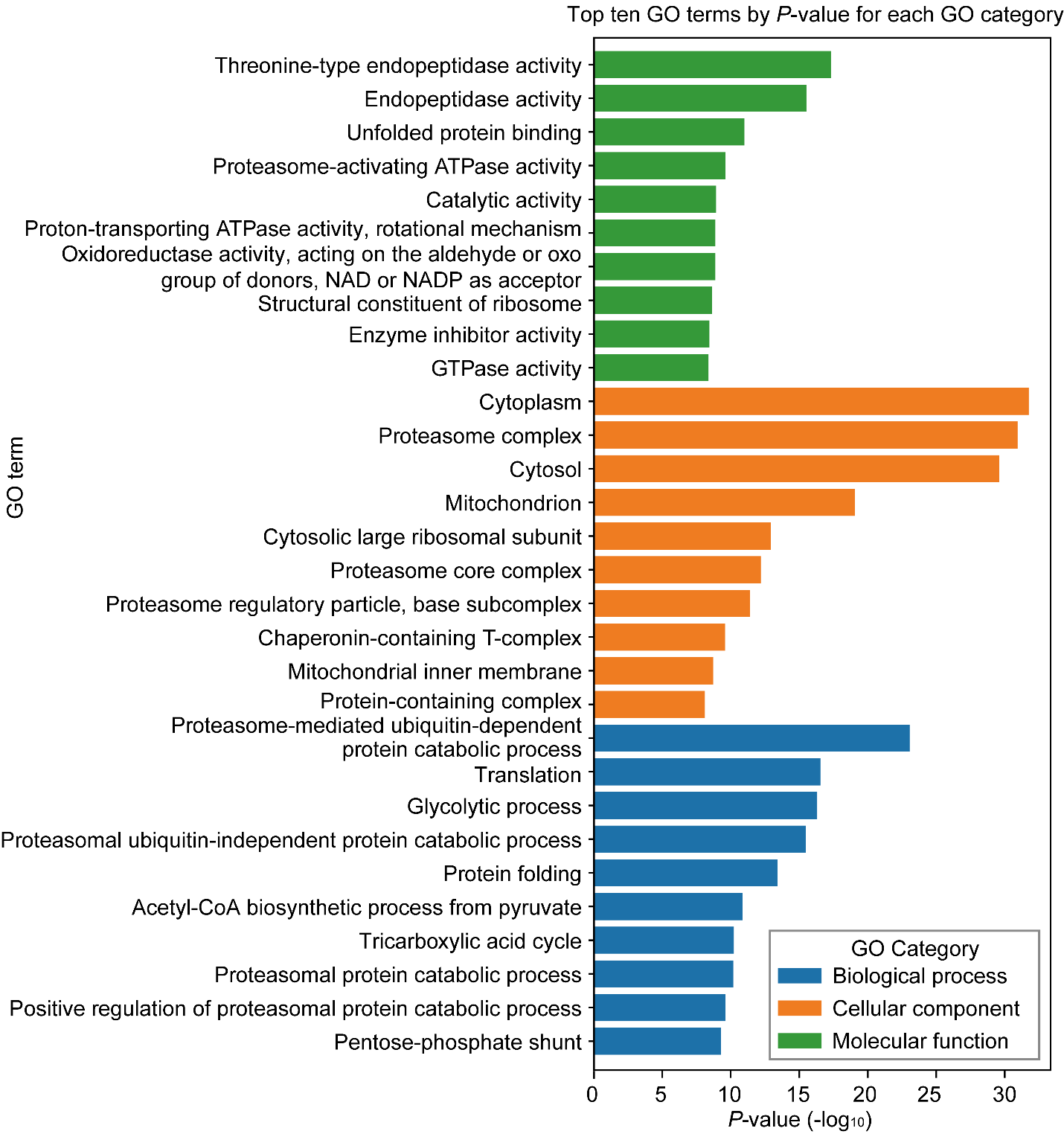


Figure S9. GO enrichment of genes in high TE group.

The gene set used for gene ontology (GO) enrichment analysis was shown in data S2. In total, 2,544 genes were used for analysis, and the bar plot showed the first ten categories of each GO category. The method was detailed in the Materials and Methods section.


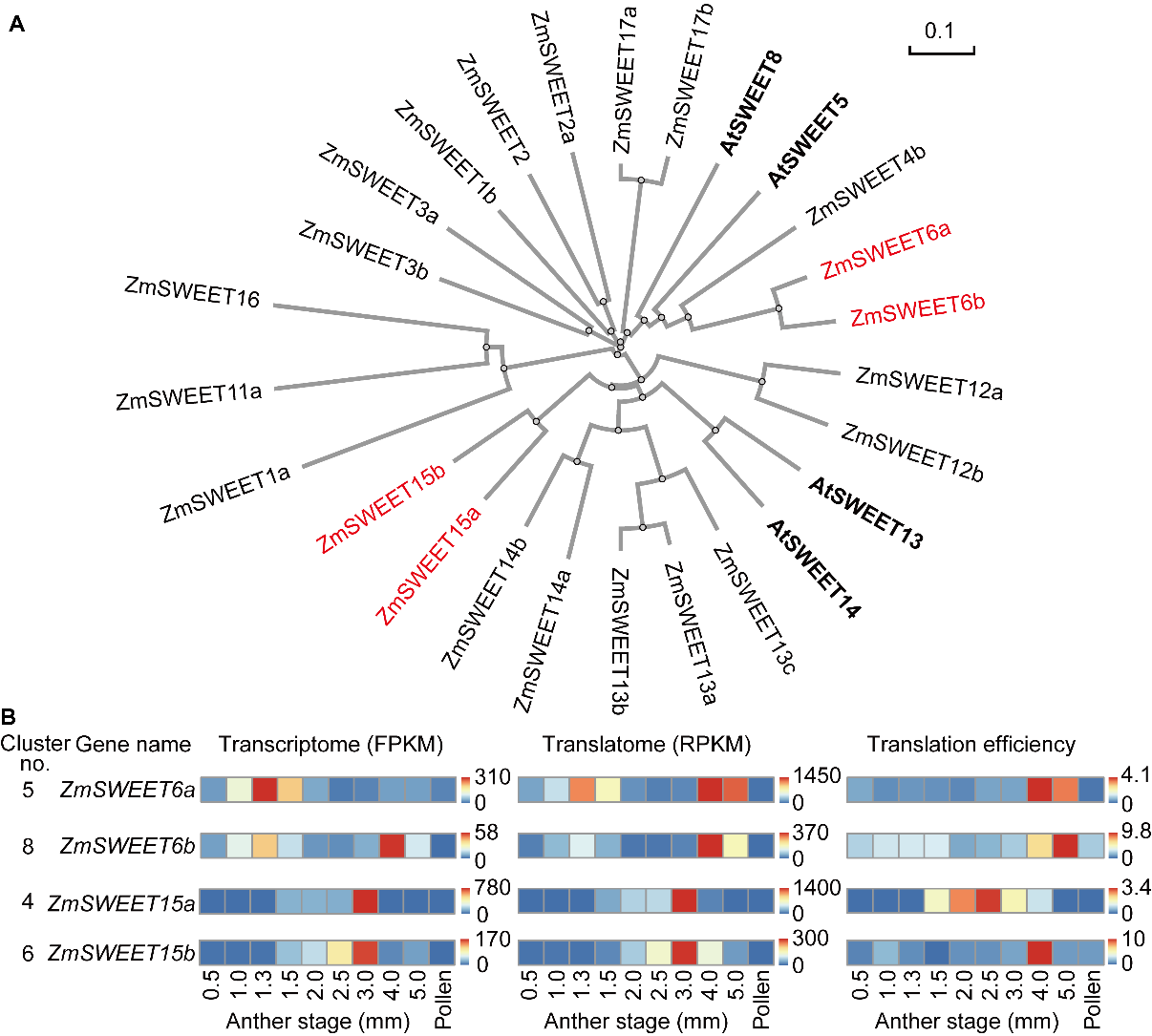


Figure S10. Phylogram of maize SWEETs and AtSWEET5/8/13/14.

A. The protein sequences of maize or *Arabidopsis* SWEET genes (*AtSWEET5/8/13/14*) were downloaded from MaizeGDB (https://www.maizegdb.org/) or TAIR (https://www.arabidopsis.org/), respectively. Multiple protein sequences alignment was performed in the online tool Muscle (https://www.ebi.ac.uk/jdispatcher/msa/muscle?stype=protein) using the ClustalW program.

B. The expression levels of four maize *SWEETs* (*ZmSWEET6a/b* and *ZmSWEET15a/b*) in transcriptome (FPKM, fragments per kilobase of transcript per million mapped reads) and translatome (RPKM, reads per kilobase of transcript per million mapped reads). The clustering information of genes is derived from Figure 2A.


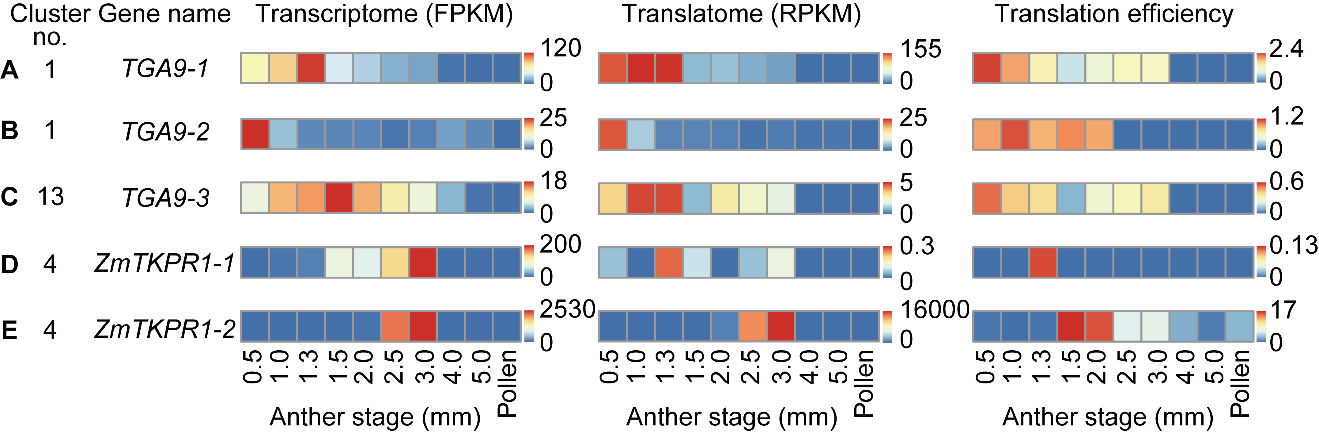


Figure S11. The transcript level, translation level and translation efficiency of *TGA9-1/2/3* and *ZmTKPR1-1/2*.

Each gene is shown with its corresponding cluster assignment, as defined in Figure 2A of the main text.


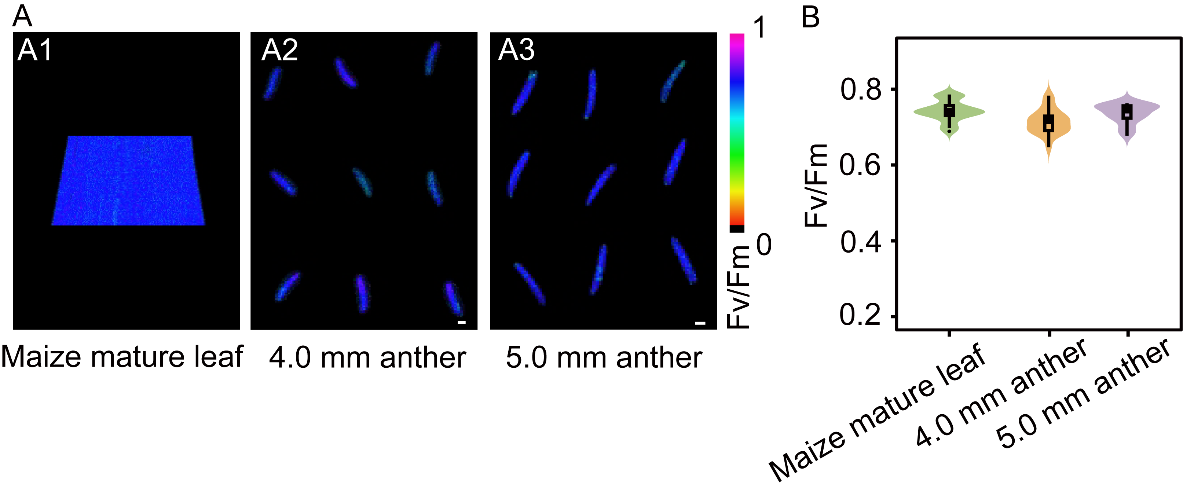


Figure S12. The photosynthetic potential of maize anthers is comparable to that of mature leaves.

A and B. Photosynthetic activities of maize mature leaf (A1) and anthers at 4.0- (A2) and 5.0-mm (A3) anthers were measured and compared by using chlorophyll fluorescence Fv/Fm characteristics (B).


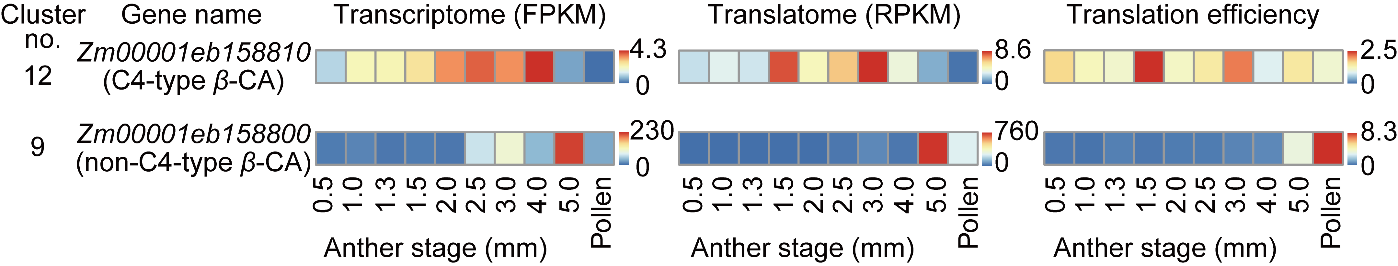


Figure S13. The transcript level, translation level and translation efficiency of two *β*-CA genes.

Each gene is shown with its corresponding cluster assignment, as defined in Figure 2A of the main text.


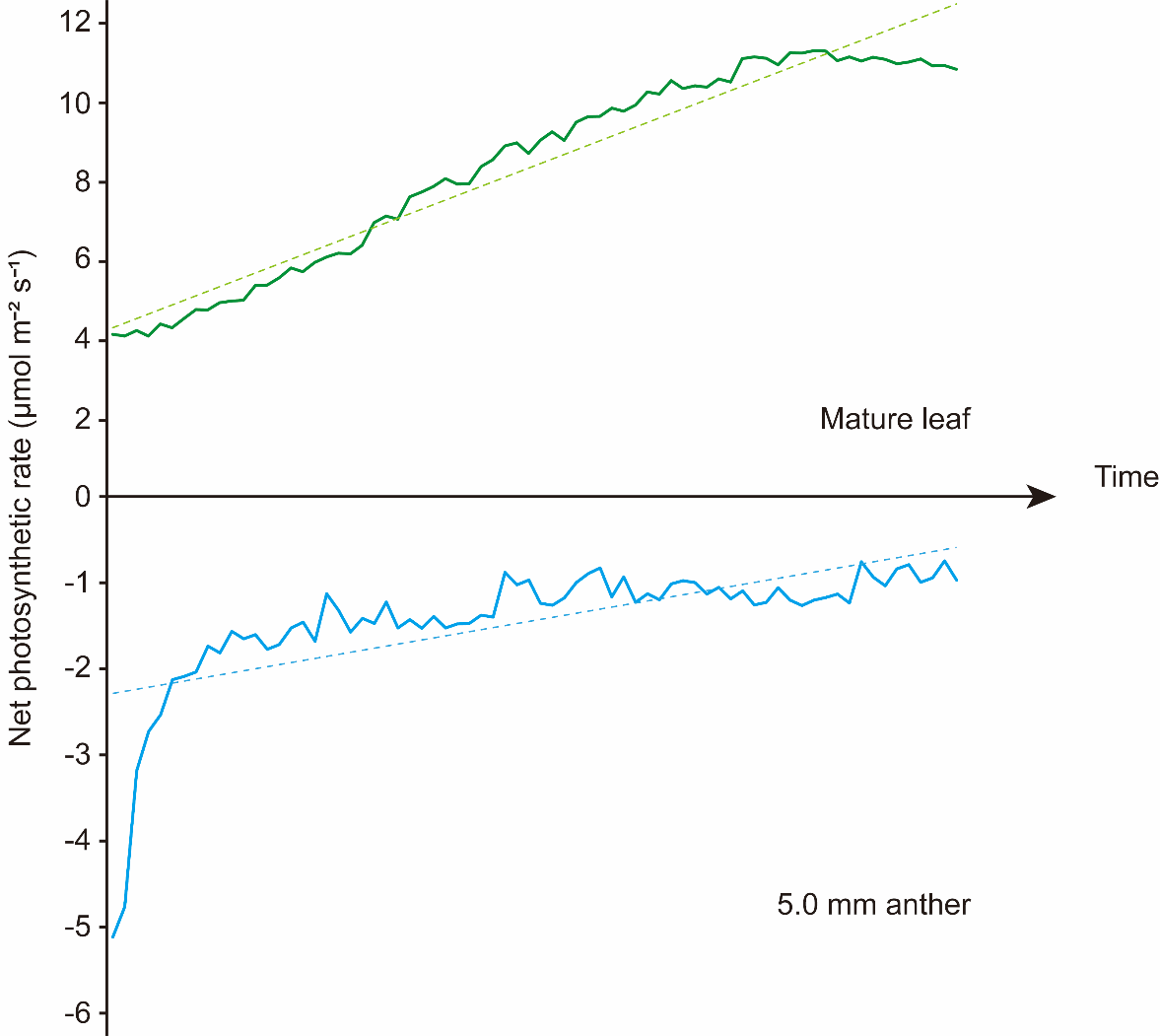


Figure S14. Net photosynthetic rate of mature leaves and anthers of maize.

The net photosynthetic rate of mature leaves (Top panel) and anthers (Bottom panel) was measured by Chlorophyll fluorescence-imaging-gas exchange simultaneous measurement system (GFS-DUAL).


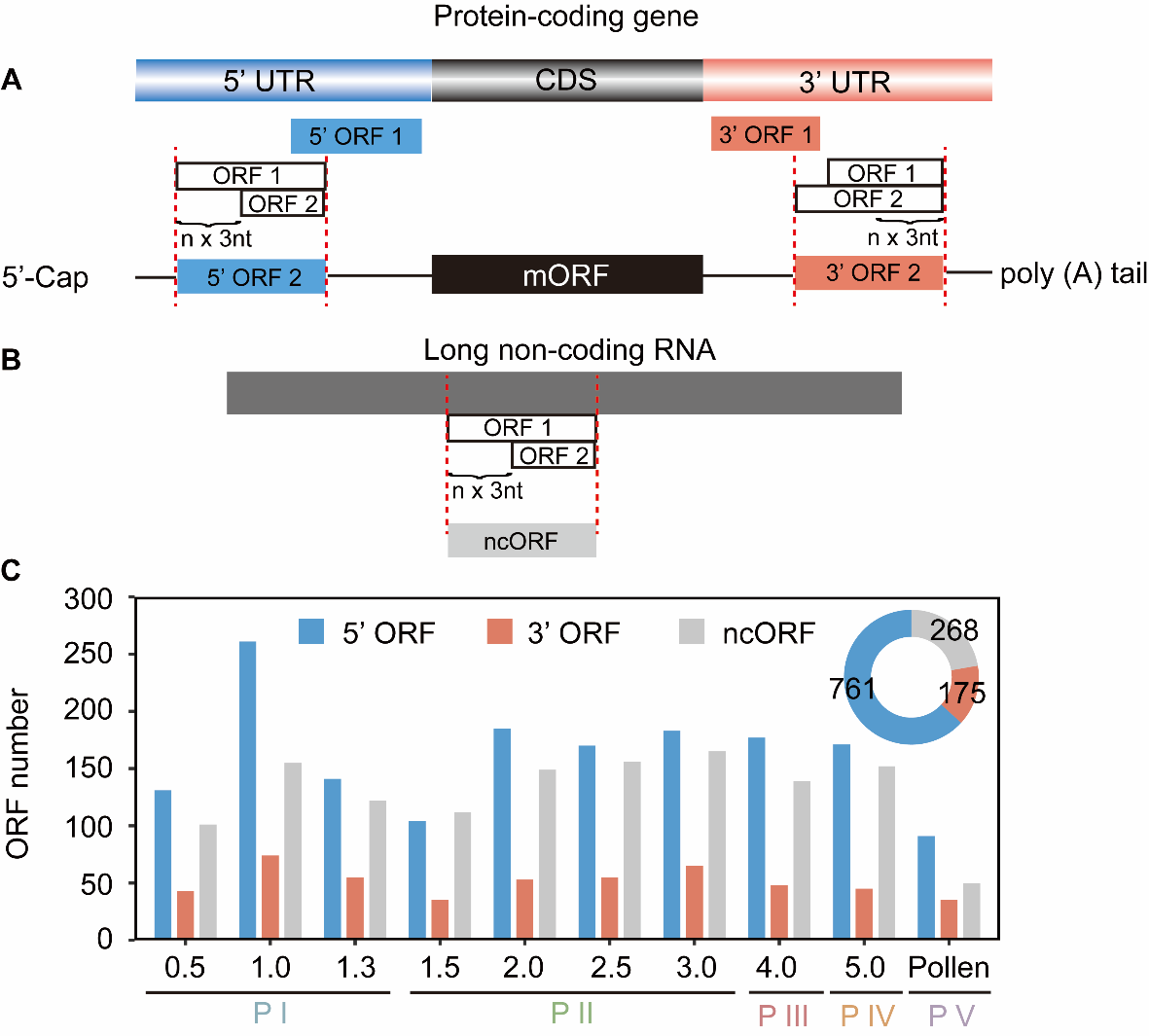


Figure S15. Identification of 5′, 3′ ORFs and ncORFs.

A. Schematic diagram showing open reading frames (ORFs) in protein-coding and adjacent regions of genes. ORFs located in untranslated regions (UTRs) that overlap sequences with 3-nt periodicity were merged into a single 5′ or 3′ ORF. UTR, untranslated region; CDS, coding sequence; ORF, open reading frame; mORF, main ORF; nt, nucleotide.

B. Schematic diagram showing ncORF detected in the long non-coding RNA. ORFs that share the same stop codon and exhibit 3nt periodicity in their non-overlapping sequences are defined as a single ncORF. ncORF, non-coding ORF. nt, nucleotide.

C. Numbers of translated ORF types identified genome-wide by ribosome profiling of samples representing ten stages of maize anther development. Phase divisions of anther development are shown in Figure 1. P, phase.


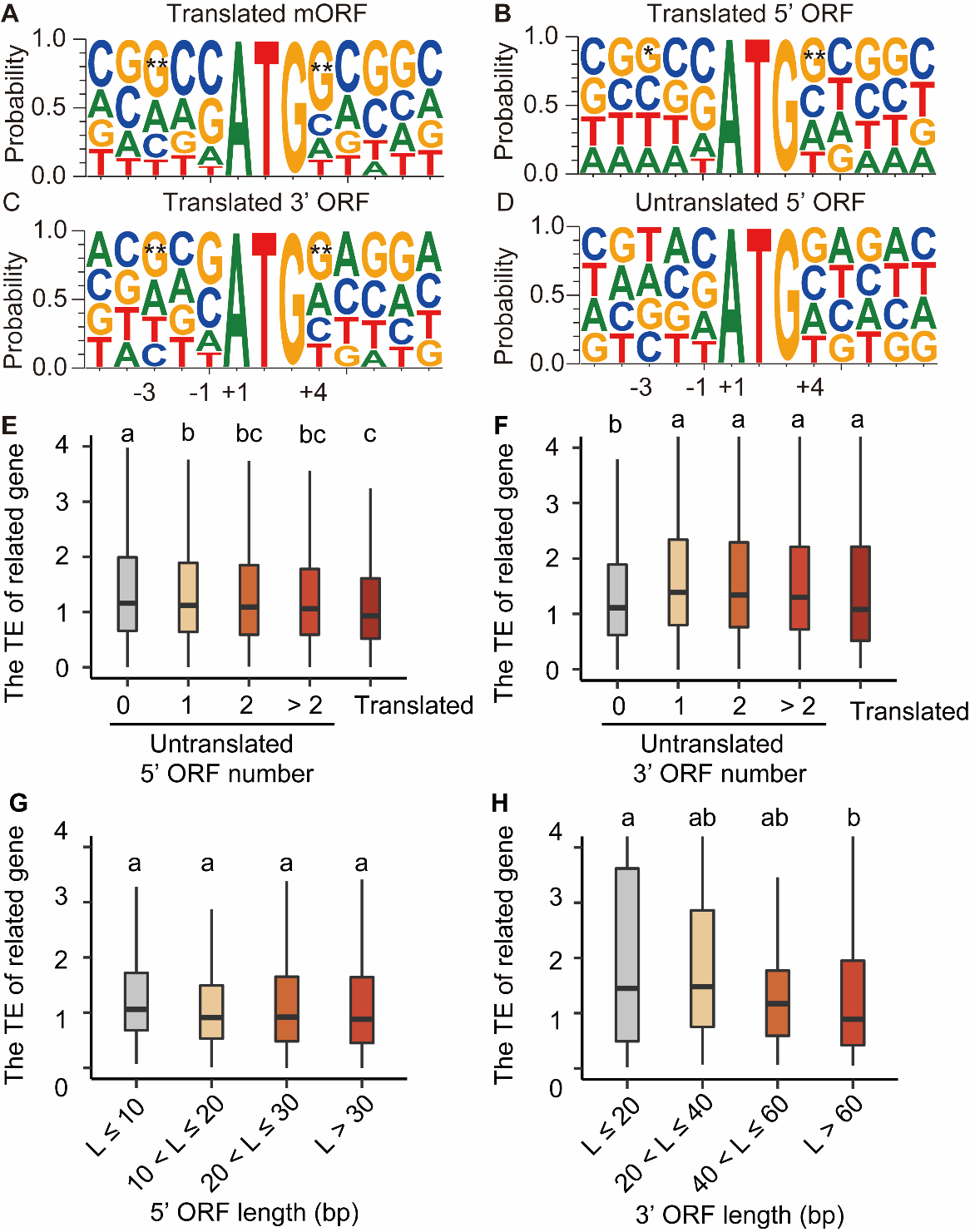


Figure S16. Gene transcription efficiency responds differently to different characteristics of 5′ or 3′ ORFs.

A to D. Kozak sequence analysis of translated main open reading frames (mORFs) of protein coding gene (A), translated 5′ ORFs (B) and 3′ ORFs (C), and untranslated 5′ ORFs (D).

E. Box plot shows the relationship between the different number of non-translated 5′ ORFs or translated 5′ ORFs and the translation of their corresponding gene.

F. Box plot shows the relationship between the different number of non-translated 3′ ORFs or translated 3′ ORFs and the translation of their corresponding gene.

G and H. The box plots show the relationship of 5′ ORFs (G) or 3′ ORFs (H) in different length and the TE of their corresponding genes. L denotes the length of 5′ ORFs (G) or 3′ ORFs (H). bp, base pair. TE, translation efficiency. Different lowercase letters indicate significant differences (* *P* < 0.05) determined by Tukey’s honestly significant difference (HSD) test.


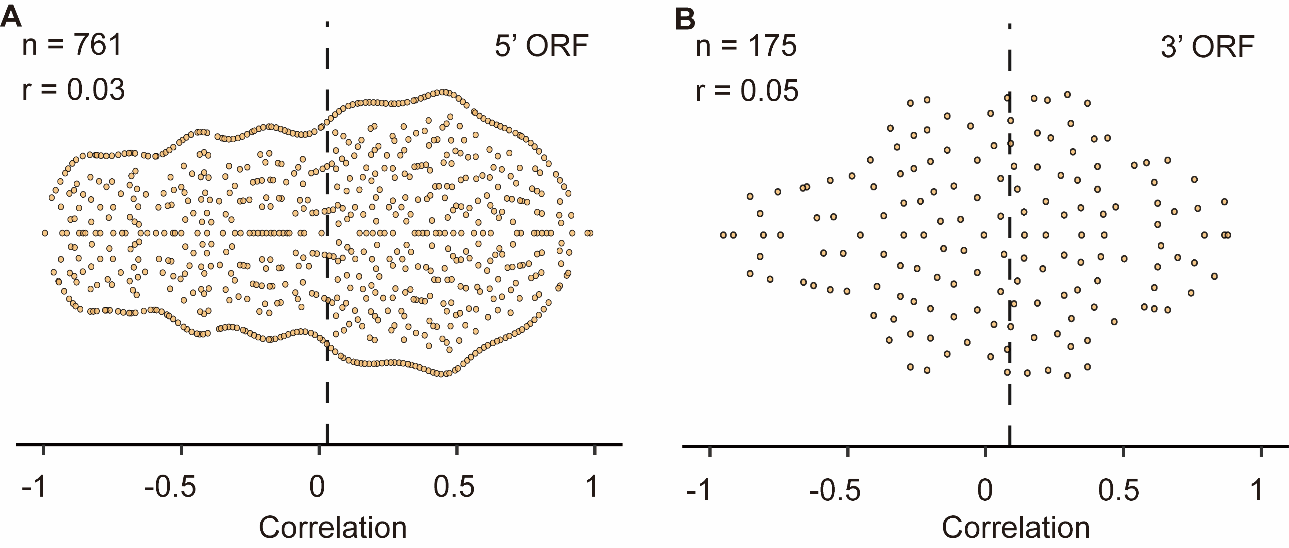


Figure S17. Beeswarm dot plot showing the correlation between translated 5′ ORFs (A) or 3′ ORFs (B) and the TE of their corresponding genes.

TE, translation efficiency. n denotes the number of 5′ ORF (A), or 3′ ORF (B). r denotes the correlation. ORF, open reading frame.


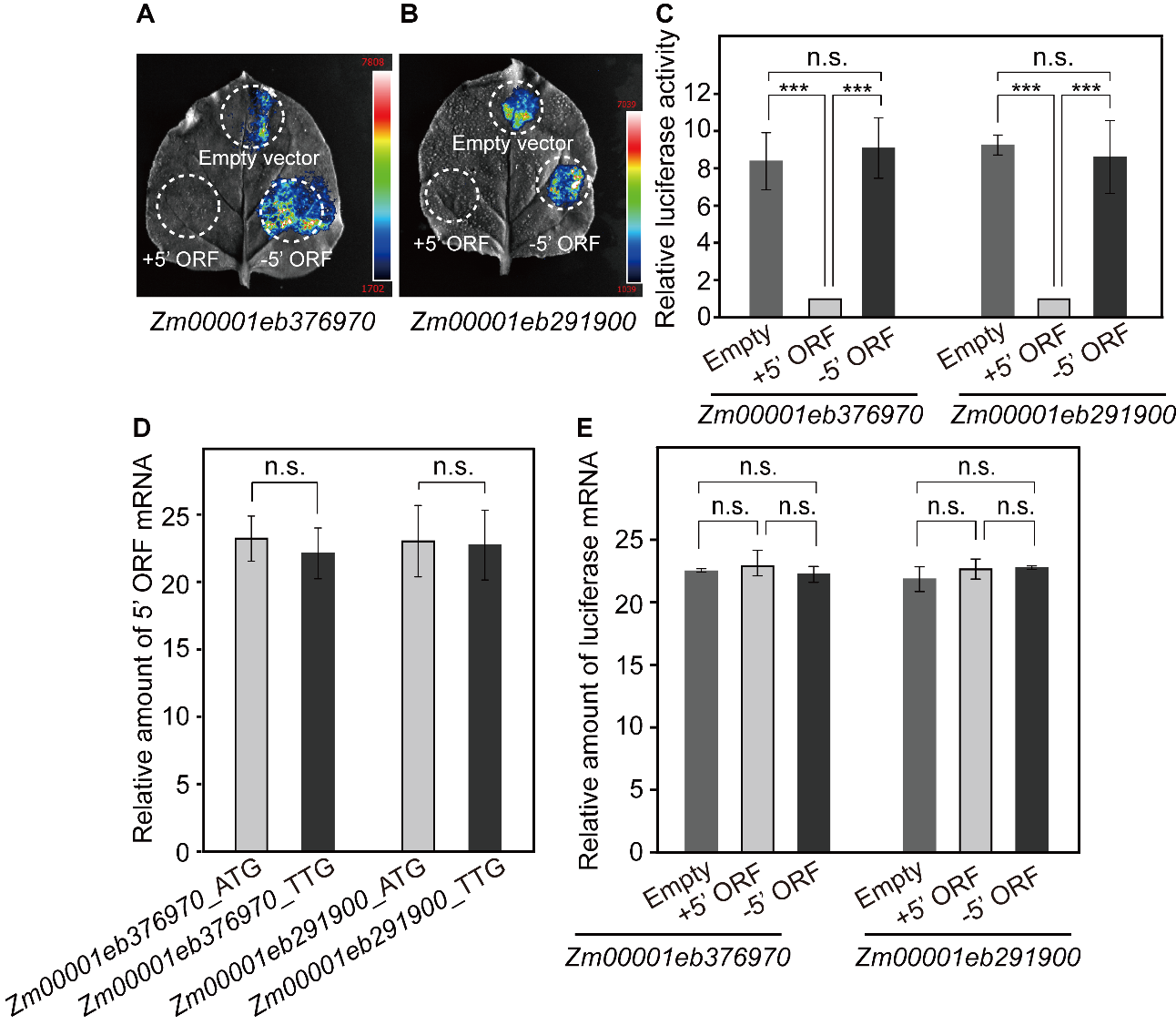
Figure S18. The inhibition of main ORF translation by 5′ ORF is not at the transcriptional level.

A and B. Inhibitory effect of 5′ ORFs in the 5′ UTR on main ORF translation of cognate gene. Images show the 5′ ORFs of *Zm00001eb376970* (A) and *Zm00001eb291900* (B) have the inhibitory effect on their mORFs. The construction of fusion vectors was shown in Figure 5C. The white dotted circles mark the injection areas on the leaves and are utilized for detecting the mRNA levels of the 5′ ORF and mutated 5′ ORF in the empty vector, +5′ ORF vector, and -5′ ORF vector, as well as measuring the LUC mRNA level and LUC fluorescence intensity.

C. The relative luciferase activity of empty vector, +5′ ORF vector and -5′ ORF vector in panel A and B. n.s. indicates no significant difference. *** denotes *p* < 0.001.

D. The mRNA level of 5′ ORF and mutated 5′ ORF for *Zm00001eb376970* and *Zm00001eb291900*. n.s. indicates no significant difference.

E. The LUC mRNA levels of empty vectors, +5′ ORF vectors, and -5′ ORF vectors for *Zm00001eb376970* and *Zm00001eb291900*. n.s. indicates no significant difference. *t*-test, n = 3.


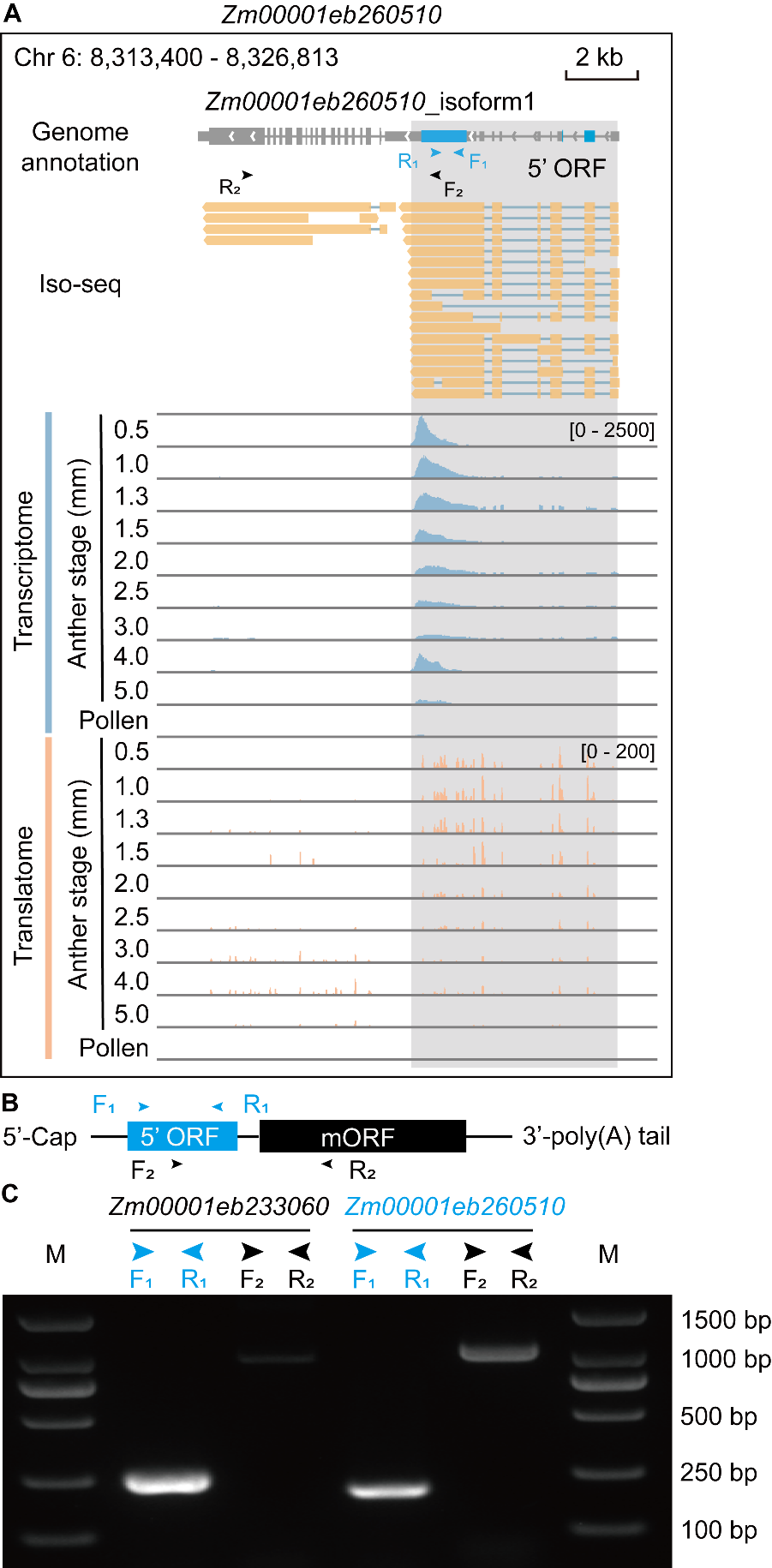


Figure S19. The 5′ ORF identified as a new isoform of *Zm00001eb260510* and expressed independently.

A. The isoform of gene structure and ribosome footprints in maize anthers are depicted for *Zm00001eb260510*. The shaded areas represent the coverage region of transcriptome and translatome reads. The numbers in the reads area indicate the maximum reads range of transcriptome of translatome. The yellow boxes or yellow boxes connected by light blue lines in Iso-seq panel represent identified isoforms of *Zm00001eb260510*. Iso-seq represents the full length transcriptome.

B. Schematic diagram of 5′ ORF independent amplification.

C. Agarose gel electrophoresis showed that 5’ ORF of *Zm00001eb260510* could be independently transcribed in maize anther by using the cDNA library of 3.0-mm anthers. Genome annotations of *Zm00001eb260510* were downloaded from MaizeGDB (https://www.maizegdb.org/). The blue boxes represent the translated 3′ ORF identified by Ribosome profiling. The blue arrowheads indicate the primers in 5′ ORF region, and the black arrowheads represent that the primers span the 5′ ORF and mORF region. Chr, chromosome. F represents the forward primer, while R denotes the reverse primer. ORF, open reading frame. mORF, main ORF. M denotes the DNA marker (BM401-02, TRANS, China).


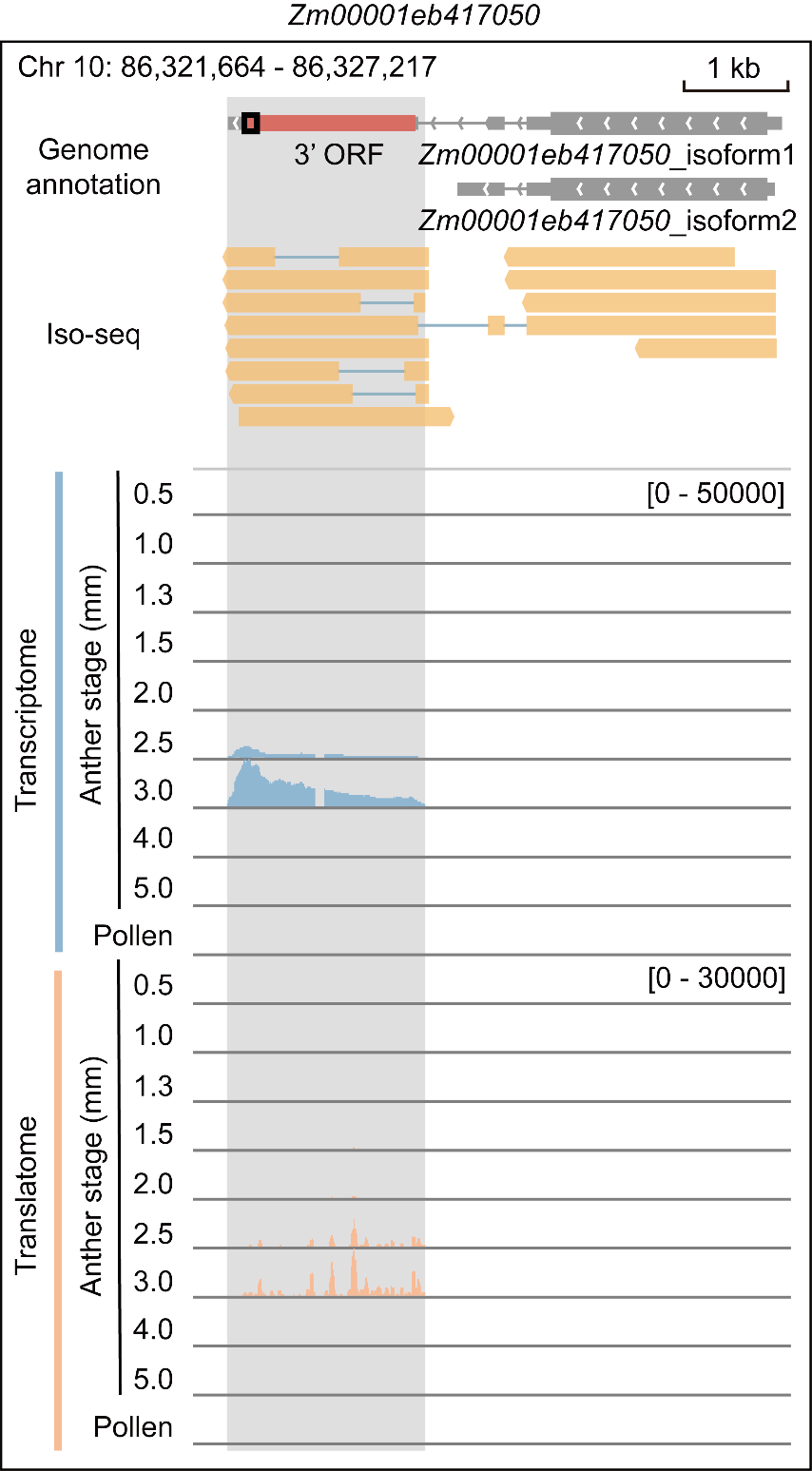


Figure S20. The 3′ ORF of *Zm00001eb417050* was dominantly expressed during the 2.5- and 3.0-mm stages as an isoform defined by Iso-seq.

The genome annotation for *Zm00001eb417050* was downloaded from MaizeGDB (https://www.maizegdb.org/). The vermilion box represents the translated 3′ ORF identified by ribosome profiling. The shaded area represents the coverage region of transcriptome and translatome reads. The yellow boxes or yellow boxes connected by light blue lines in Iso-seq panel represent identified isoforms of *Zm00001eb417050*. The numbers in the reading area indicate the maximum reads range of transcriptome of translatome. The black box represents the sequence of the mutation in both the wild type and *apv1* mutant (Figure 5, D and E). Chr, chromosome. ORF, open reading frame. Iso-seq represents the full-length transcriptome.


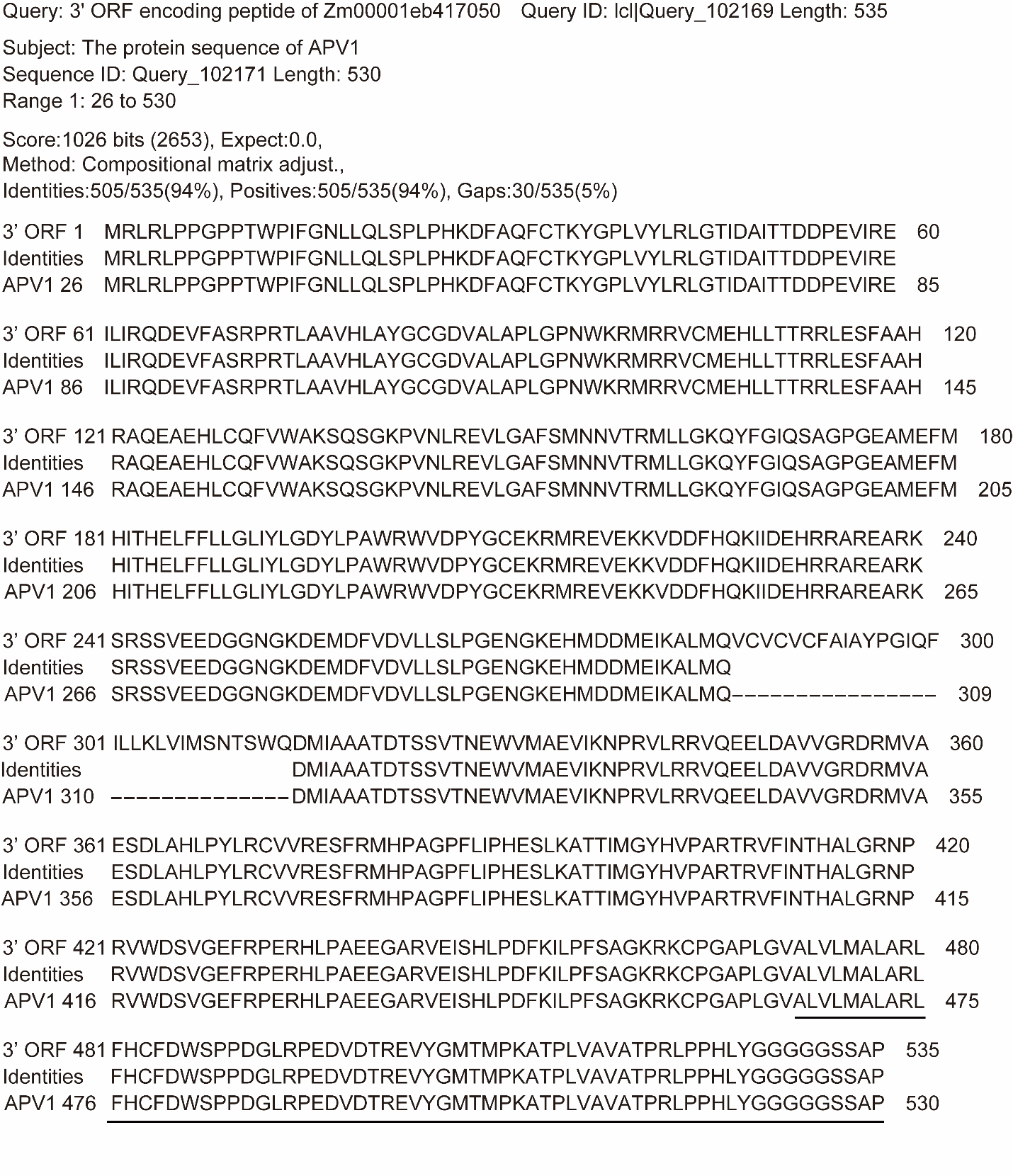
Figure S21. Sequence alignment of the Zm00001eb417050_3' ORF encoding peptide with the protein sequence of *APV1*.

The protein sequence of APV1 was downloaded from MaizeGDB (https://www.maizegdb.org/). Underline represents the fragments deletion in the *apv1* mutant. The numbers at both ends of the protein sequence represent the positions of residues in 3' ORF or APV1. The dotted line represents the sequence difference between the APV1 and the peptide encoded by the Zm00001eb417050_3' ORF.


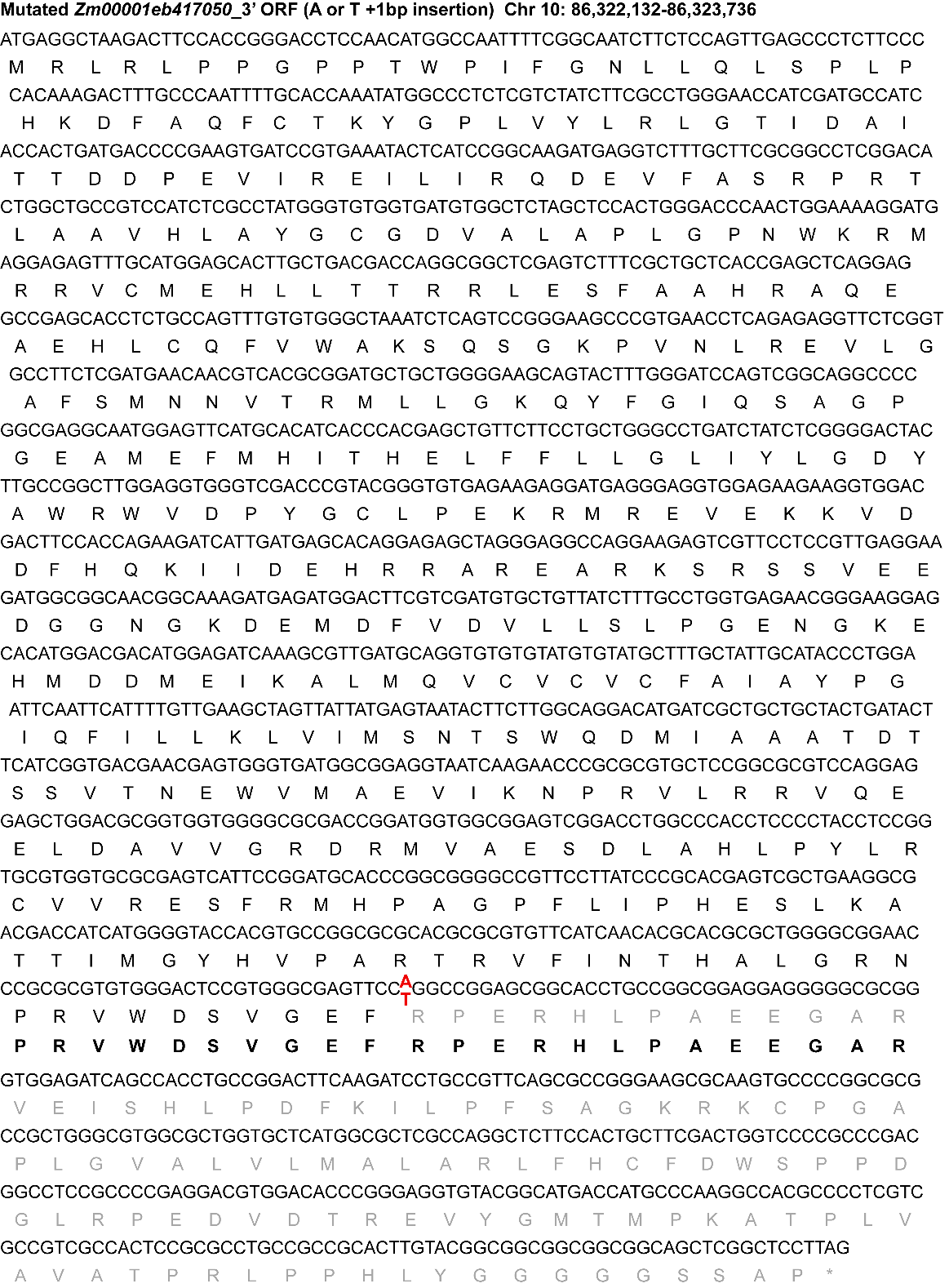


Figure S22. The +1 bp insertion in the 3′ ORF of *Zm00001eb417050* caused a frameshift mutation in its encoded peptide.

The red capital letter T represents the inserted 1bp, and the gray letters represent the frameshift mutation sequence. The protein-coding sequence of *GRMZM5G830329* near the +1 bp insertions is highlighted in bold to facilitate comparison with the 3′ ORF encoded sequence. The protein sequence of APV1 was downloaded from MaizeGDB (https://www.maizegdb.org/).


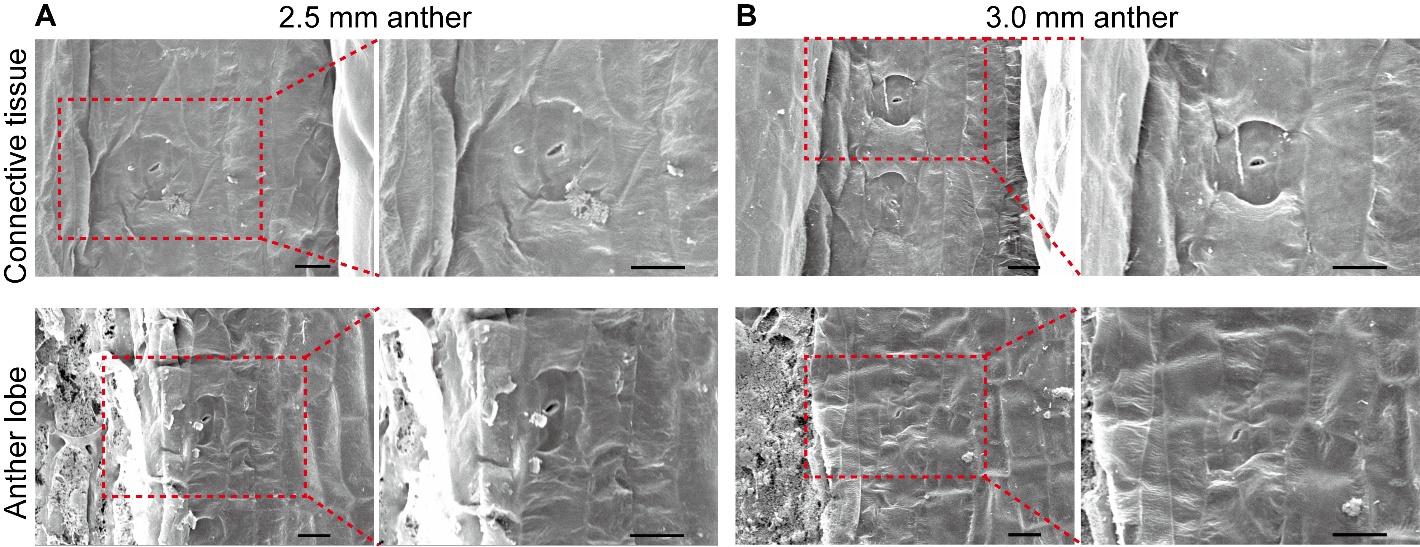


Figure S23. Stomata on the surface of anther at the 2.5– and 3.0–mm stages.

Open stomata were also observed on the anther connective tissue and lobe surface at the 2.5– (A) and 3.0–mm (B) stages. Scale bar, 10 μm.

Supplementary dataset

**Sheet 1.**

The expression information of all expressed genes at all stages.

**Sheet 2.**

The expression information of the genes belonging to the high TE group.

**Sheet 3.**

The expression information of genes belonging to the low TE group.

**Sheet 4.**

The gene list of the genes with translation efficiency fluctuating between stages.

**Sheet 5.**

The ORFs detected in the UTR of genes or genomic non-coding region.

**Sheet 6.**

ORFs overlapping independent isoforms detected by Iso-seq data.

**Sheet 7.**

Small peptides detected by mass spectrometry of 5' and 3' ORFs.

**Sheet 8.**

List of primers used in this article.
